# Testing the Dry Refuge Model: Paleoecological Insights From Late Pleistocene Gomphotheres in Ecuador

**DOI:** 10.1002/ece3.74099

**Published:** 2026-08-02

**Authors:** Erwin González‐Guarda, Iván Ramírez‐Pedraza, Carlos Tornero, Lidiane Asevedo, Natalia Villavicencio, Sebastián Escobar, José Luis Román, Melissa Hidalgo, Esteban Benalcázar, Florent Rivals

**Affiliations:** ^1^ Instituto de Ciencias de la Ingeniería Universidad de O'Higgins Rancagua Chile; ^2^ Institut Català de Paleoecologia Humana i Evolució Social (IPHES‐CERCA) Tarragona Spain; ^3^ Departament de Biologia Evolutiva, Ecologia i Ciències Ambientals, Secció de Zoologia i Antropologia Biològica Universitat de Barcelona Barcelona Spain; ^4^ Institut d'Arqueologia de la Universitat de Barcelona Barcelona Spain; ^5^ Department of Prehistory Autonomous University of Barcelona (UAB) Bellaterra Spain; ^6^ Departamento de Estratigrafia e Paleontologia, Faculdade de Geologia Programa de Pós‐Graduação em Geociências, Universidade do Estado do Rio de Janeiro Rio de Janeiro Brazil; ^7^ Instituto de Geografía, Facultad de Historia, Geografía y Ciencia Política Pontificia Universidad Católica de Chile Santiago Chile; ^8^ Biología Integrativa, Facultad de Ciencias Biológicas Pontificia Universidad Católica de Chile Santiago Chile; ^9^ Grupo de Investigación en Ecología y Evolución en los Trópicos‐EETrop Universidad de Las Américas Quito Ecuador; ^10^ Departamento de Biología, Facultad de Ciencias Escuela Politécnica Nacional Quito Ecuador; ^11^ Terra Ignota Ñuñoa Región Metropolitana Chile; ^12^ ICREA Barcelona Spain; ^13^ Universitat Rovira i Virgili (URV), Departament d'Història i Història de l'Art Tarragona Spain

**Keywords:** carbon, dental microwear, oxygen, paleoenvironment, South America, teeth

## Abstract

Paleoenvironmental records indicate that Neotropical regions near the Equator experienced reduced rainfall during the Last Glacial Maximum (LGM). However, some records show conflicting environmental signals. To address these discrepancies, previous studies have proposed two demographic/genetic scenarios for cloud forests based on contrasting precipitation regimes. Here, we apply a multiproxy approach to extinct proboscideans, commonly referred to as gomphotheres, from Ecuador to assess the extent to which wet‐ and dry‐forest models shaped environmental conditions on the coast and in the Inter‐Andean Valley during the Late Pleistocene. Fossil molars were analyzed using stable isotope analysis, dental microwear analysis, and radiocarbon dating. Radiocarbon dates obtained from the sampled gomphotheres span *ca*. 31,820–23,830 cal yr. BP. Enamel bioapatite δ^13^C values, ranging from −14.82‰ to −0.78‰, indicate that these proboscideans occupied a broad range of environments, although the overall isotopic signal points to a predominance of more open and relatively dry habitats. Dental microwear data support this interpretation, indicating substantial grass consumption. Reconstructed meteoric water δ^18^O values derived from enamel bioapatite reveal a clear altitudinal effect on gomphothere distribution. Moreover, these values are comparable to those recorded at present‐day meteorological stations in Ecuador. We therefore infer that, at least during the lifetimes of the sampled individuals, there were no major differences in the δ^18^O signature of atmospheric circulation between the Late Pleistocene and today in this region. Although substantial chronological gaps remain in paleoenvironmental reconstructions, our results suggest that processes consistent with the Dry Refuge Model (DRM) had a strong influence on Ecuadorian landscapes during the Late Pleistocene. In this scenario, cloud forests were displaced and compressed into refugia due to the combined effects of aridity and cooling.

## Graphical abstract

We present paleontological evidence that may prove the Dry Refuge Model in Ecuador during the Late Pleistocene.

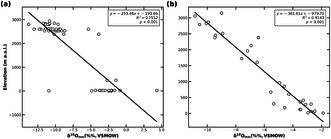

## 1 Introduction

Proboscideans of the family Gomphotheriidae are among the most conspicuous components of Quaternary paleontological deposits in South America. Recent taxonomic revisions recognize two species on the continent: *Notiomastodon platensis* (Ameghino 1888) and *Cuvieronius hyodon* (Fischer 1814) (Mothé and Avilla 2015; Mothé et al. 2017). South American gomphotheres are widely recognized for their ecological plasticity and their ability to occupy a broad range of ecosystems. Multiproxy studies indicate that these populations were not restricted to a single biome, but instead exhibited broad environmental tolerance, ranging from grasslands to dense forests (González‐Guarda et al. 2018). This dietary flexibility expressed through varying degrees of grazing, browsing, and mixed‐feeding behavior suggests that gomphotheres acted as ecological generalists capable of adjusting their feeding strategies to local resource availability under the climatic fluctuations of the Late Pleistocene (Asevedo et al. 2020).

This ecological flexibility makes gomphotheres particularly useful for paleoenvironmental reconstruction. Because their diets are expected to reflect the vegetation available in the environments they inhabited, gomphothere fossil remains can provide direct faunal evidence for assessing spatial variation in vegetation structure and environmental conditions (Asevedo et al. 2020; González‐Guarda et al. 2018). This interpretation is supported by multiple lines of evidence, including biomechanical analyses of *N. platensis* (Benalcázar 2025) and the correlation between gomphothere diets, particularly browsing behavior, and vegetation structure, as inferred from pollen‐based estimates of tree biomass along a 1500‐km latitudinal gradient in central Chile (González‐Guarda et al. 2024, 2025). Recently, Ziegler et al. (2025; preprint) proposed that northern Andean environments may have functioned as refugia that buffered gomphothere populations against marked environmental change, acting as relatively stable ecological corridors. In this context, the Ecuadorian gomphothere record has particular relevance for understanding landscape evolution and environmental heterogeneity in northwestern South America.

The Late Pleistocene environmental history of the Neotropics remains debated (Van der Hammen and Hooghiemstra 2000). The forest refugia hypothesis, originally formulated by Haffer (1969), proposes that Late Pleistocene climatic oscillations caused cyclical changes in the extent of Neotropical forests, leading to the persistence of plant communities in stable habitat patches that acted as refugia during more arid glacial periods (Hewitt 2004; Carnaval et al. 2009; Fontes et al. 2017). In contrast, alternative perspectives suggest a higher degree of stability in tropical forests throughout glacial cycles (Hostetler and Mix 1999; Beerling and Mayle 2006; Cheng et al. 2013), implying that changes in precipitation had a relatively limited effect on the continuity of forest cover. These opposing views have sustained debate over whether cloud forests, defined here as tropical and subtropical montane forests characterized by persistent humidity, were fragmented into refugia during the LGM.

**FIGURE 1:**
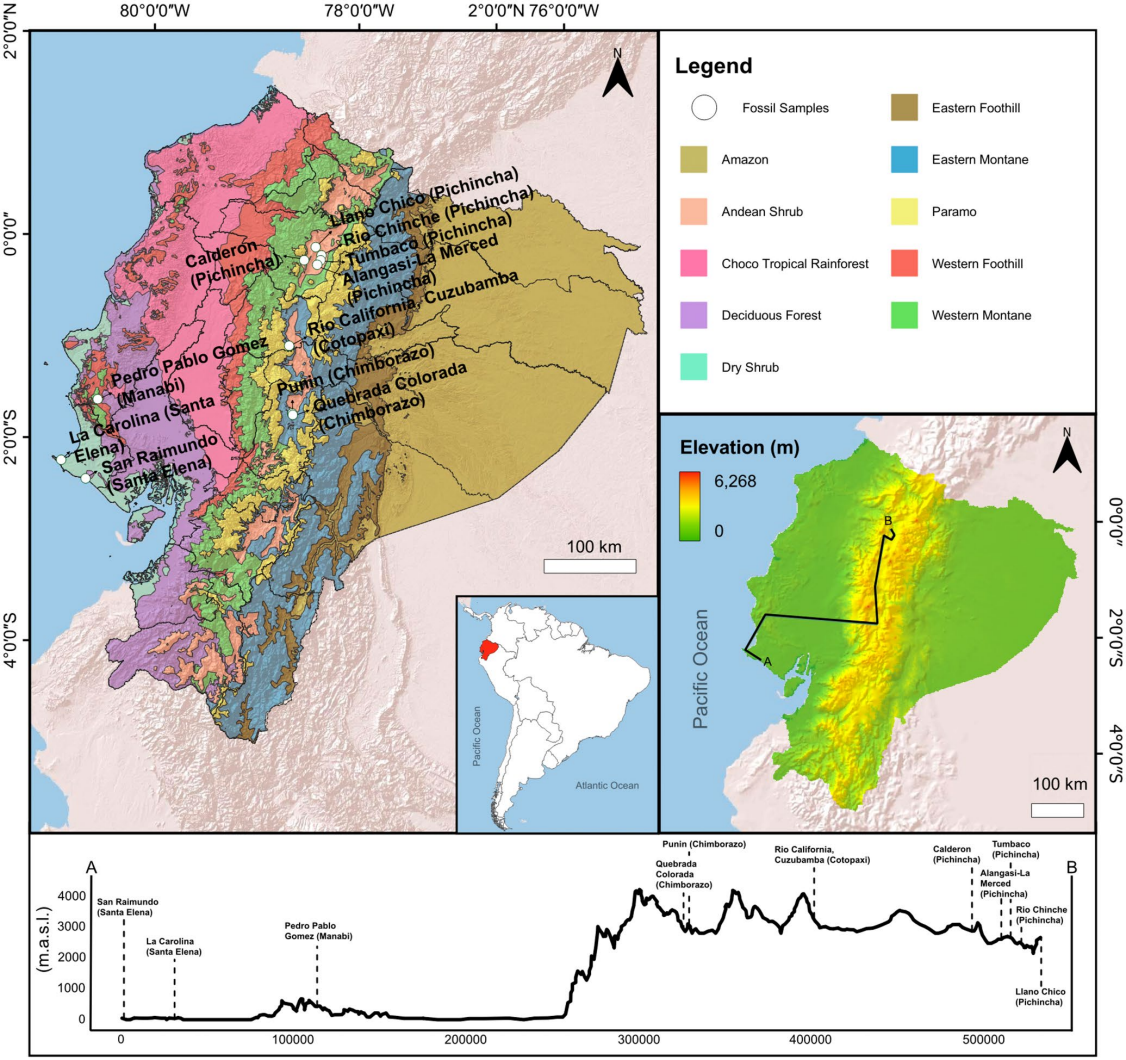
Geographic distribution of gomphothere specimens analyzed in this study in Ecuador, indicating the locations of fossil sites and the distribution of modern vegetation types (Ministerio de Ambiente del Ecuador 2013).

In the western flank of the Ecuadorian Andes (Figure 1), the Late Pleistocene distribution of tropical forest refugia remains unresolved. Based on the present‐day plant distribution patterns, lowland tropical forests have been hypothesized to have persisted during glacial stages within the Chocó refuge, located along the northwestern Pacific coast of Ecuador and the Pacific coast of western Colombia (Prance 1982). Furthermore, premontane rainforests (1000–1800 m a.s.l.) may also have acted as refugia, as they are found in the western foothills of the Andes Mountains of Ecuador. In this context, two more specific models have been proposed (Ramírez‐Barahona and Eguiarte 2013). The first, the moist forest hypothesis, suggests that glacial cooling promoted the downslope displacement of premontane and montane Neotropical rainforests to lower altitudes (Colinvaux et al. 1997). However, the availability of moisture in adjacent lowlands may have been a key factor controlling the extent and direction of this displacement of vegetation (Ramírez‐Barahona and Eguiarte 2013). In this scenario, the Andean foothills of northwestern Ecuador may not have acted as refugia during the Pleistocene, as premontane rainforests could instead have migrated downslope toward the northern coast (Hooghiemstra and van der Hammen 1998).

Second, the dry forest hypothesis predicts the isolation of forests at mid‐altitudes (Ramírez‐Barahona and Eguiarte 2013). Because the lowland areas of southwestern Ecuador remained relatively dry during the Pleistocene (Heusser and Shackleton 1994), the adjacent Andean foothills could have supported premontane rainforests during glacial cycles, as they were unable to migrate to the arid lowlands (Escobar et al. 2021). In this scenario, cloud forests were displaced and confined to refugia by the combined effects of aridity and cooling and later expanded to recolonize their former range as warmer and wetter conditions returned (Ramírez‐Barahona and Eguiarte 2013). These models generate contrasting paleoenvironmental expectations. Under the moist forest model, lowland and mid‐elevation environments should have remained relatively humid and forested, ranging from scrubland to dense forest. Under the dry forest model, more open and arid landscapes, ranging from grasslands to scrubland, would be expected. Despite the importance of Ecuador for testing these models, Late Pleistocene paleoenvironmental records remain scarce. At the same time, Ecuador preserves an abundant fossil record of gomphotheres across a broad altitudinal gradient, from near sea level, at approximately 8 m a.s.l., to the high Andes, at approximately 3000 m a.s.l. This makes gomphotheres a valuable proxy for assessing environmental variation across the coast and the Inter‐Andean Valley. Here, we use a multiproxy approach combining stable isotope analysis, dental microwear analysis, and radiocarbon dating to evaluate the influence of moist‐ and dry‐forest models on the environments occupied by gomphothere populations in Ecuador during the Late Pleistocene. Specifically, we investigate dietary and paleoenvironmental patterns to infer the predominant vegetation types present on the coast and in the Inter‐Andean Valley at the temporal scale represented by the sampled individuals.

We hypothesize that the habitats occupied by Ecuadorian gomphotheres during the Late Pleistocene were more arid and open than present‐day environments on the western slope of the Andes, as a consequence of the cold Humboldt Current along the Pacific coast and the reduced moisture transport from the Amazon Basin and Atlantic Ocean during glacial phases (Vuille et al. 2000). If the dry forest model applies to the Ecuadorian record, we expect isotopic and dental microwear evidence to indicate a predominance of open, relatively dry habitats and a higher contribution of grasses or abrasive vegetation to gomphothere diets. Conversely, if the moist forest model better explains the environmental context of these populations, we expect evidence for more closed, humid environments and a greater contribution of browse‐dominated resources.

## 2 Material and Methods

### 2.1 Material

Molars of the extinct proboscideans *N. platensis* and *C. hyodon* were analyzed (Sánchez et al. 2004; Mothé and Avilla 2015; Mothé et al. 2017). In total, 43 bulk enamel samples from gomphothere molars were analyzed for stable carbon and oxygen isotopes in the structural carbonate of the bioapatite (δ^13^C and δ^18^O) (Table S1; Figure 1). In addition, carbon isotope data from 16 specimens previously published by Sánchez et al. (2004) were included in the statistical analyses. For dental microwear analysis, 46 specimens were selected (Table S2; Figure 1). To establish a chronological framework, five enamel bioapatite samples from different specimens were radiocarbon dated (Table 1). All fossil specimens are housed in the paleontological collection of the *Laboratorio de Paleontología de la Escuela Politécnica Nacional*, Quito, Ecuador (Supporting Information).

**TABLE 1:** Radiocarbon dating from the bioapatite of the dental enamel of gomphotheres from Ecuador.

Taxon	Locality	Sample code	14C lab code	14C age	+/−	Cal mean	2*σ* range
*N. platensis*	La Carolina/Santa Elena (2°13′ S, 80°55′ W)	V‐4824	UGAMS‐74813	20,500	50	24,610	24,310–24,870
*N. platensis*	Pedro Pablo Gómez/Manabí (1°37′ S, 80°33′ W)	V‐4314	UGAMS‐74814	19,860	50	23,830	23,750–23,950
*N. platensis*	La Merced/Pichincha (0°18′ S, 78°24′ W)	V‐6143	UGAMS‐74815	20,930	60	25,190	25,000–25,340
*N. platensis*	Quebrada Colorada/Chimborazo (1°46′ S/78°39′ W)	V‐1254	UGAMS‐74816	27,960	90	31,820	31,600–32,060
*N. platensis*	Río California, Cuzubamba/Cotopaxi (1°5′54.1 S/78°41′ W)	V‐5780	UGAMS‐74817	25,120	70	29,260	29,130–29,630

### 2.2 Methods

Enamel powder samples (3.5–9.5 mg) for stable isotope analysis were chemically pretreated at the Biomarkers Lab of the *Institut Català de Paleoecologia Humana i Evolució Social* (IPHES‐CERCA), following modified protocols from Koch et al. (1997) and Tornero et al. (2013). Pretreated powders were analyzed using a Thermo Kiel III carbonate device interfaced with a Thermo Finnigan MAT 253 isotope ratio mass spectrometer at the Scientific and Technological Centers of the University of Barcelona (CCiTUB), Spain. Measurement accuracy was monitored using two internal laboratory calcium carbonate standards (RC‐1 and CECC) normalized to international standards NBS18 and NBS19. A total of 16 measurements of the RC‐1 and CECC standards were performed. The accepted δ^13^C values were +2.83‰ for RC‐1 and −20.78‰ for CECC. The mean analytical precision was ±0.01‰ for both standards.

Stable isotope values follow δ‐notation δ^H^X_sample_ = [(R_sample_ − R_standard_) − 1] × 1000, where X is the element, H is the mass of the rare heavy isotope, and R is the isotope ratio (^13^C/^12^C or ^18^O/^16^O). δ^13^C and δ^18^O values are expressed relative to the Vienna PeeDee Belemnite (VPDB) standard, with δ^18^O also converted to the Vienna Standard Mean Ocean Water (VSMOW) scale using the formula: δ^18^O_SMOW_ = (1.0309 × δ^18^O_VPDB_) + 30.909.

We applied the equation $\varepsilon^{*}$ = 2.4 + 0.034 (bm) to calculate the most appropriate bioapatite enrichment as a function of body mass (Tejada‐Lara et al. 2018). Thus, an enrichment value of +15‰ ($\varepsilon_{\text{diet}-\text{bioapatite}}^{*}$) was used for gomphotheres (Asevedo et al. 2021), based on an estimated body mass of 6 tons for *N. platensis* individuals (Dantas et al. 2020). We also derived theoretical δ^13^C values for consumed plants from diet‐to‐tissue trophic discrimination studies (Table S3).

Considering Late Pleistocene atmospheric CO_2_ δ^13^C values (δ^13^C_atm_CO_2_ of −6.5‰; Tipple et al. 2010), we classified the phytodomains of gomphothere habitats following Domingo et al. (2012): (i) Mesic woodland (−13.5‰ to −8.5‰), (ii) wooded C_3_ grassland to open, and arid C_3_ grassland (−8.5‰ to −5.5‰), (iii) open vegetation area C_3_–C_4_ (−5.5‰ to −0.5‰), and open C_4_ grassland (−0.5‰ to +7.5‰).

Following Phillips (2012), a two‐source linear mixing model based on δ^13^C values was applied to estimate the proportions of C_3_ and C_4_ plants consumed by each specimen. In Equations (1) and (2), δ^13^C_1_ = −10.5‰ (C_3_) and δ^13^C_2_ = +3.5‰ (C_4_) represent the mean δ^13^C values of C_3_ and C_4_ plant consumers ($\varepsilon_{\text{diet}-\text{bioapatite}}^{*}$ = +15‰). δ^13^C_mix_ is the measured value for the gomphotheres, and *f*_1_ and *f*_2_ are the proportions of C_3_ and C_4_ resources in the diet, respectively.

(1) $$\delta^{13}C_{\mathrm{mix}}=\delta^{13}C_{1}f_{1}+\delta^{13}C_{2}f_{2}$$

(2) $$1=f_{1}+f_{2},$$

The dietary niche breadth (*B*) was calculated following Levins' index (1968), where *p*_*i*_ is the proportion of resources consumed (Equation 3). This measure was then standardized (*B*_*A*_; Equation 4) from 0 to 1, where *n* is the number of resources consumed. Values close to 0 indicate dietary specialization, whereas values close to 1 indicate a broader, more generalist dietary niche.

(3) $$B=1/\sum p_{i}^{2},$$

(4) $$B_{A}=\left(B-1\right)/\left(n-1\right)$$

The δ^18^O values of meteoric water ingested by gomphotheres were estimated from their enamel $\delta^{18}O_{{\mathrm{PO}}_{4}}$ values using a linear regression based on modern elephants, their closest living relatives. To carry out this analysis, we assumed that there were no significant differences in the water‐to‐phosphate oxygen isotope fractionation factor between extinct gomphotheres and extant elephants. The equation used to obtain the δ^18^O_meteoric water_ value for gomphotheres was: δ^18^O_meteoric water (VSMOW)_ = ($\delta^{18}O_{{\mathrm{PO}}_{4}\left(\text{VSMOW}\right)}$ − 23.3)/0.94 (Ayliffe et al. 1992). Diagenetic preservation was assessed following Iacumin et al. (1996).

For the dental microwear analysis, we followed the protocols of Solounias and Semprebon (2002) and Semprebon et al. (2016). Each molar's occlusal surface was cleaned with acetone, followed by 96% ethanol. We then created high‐resolution silicone molds (Heraeus Kulzer, PROVIL novo Vinylpolysiloxane, Light C.D. 2 regular set) and produced transparent casts with clear epoxy resin (C.T.S. Spain, EPO 150 + K151). Enamel microwear features were examined using a Zeiss Stemi 2000C stereomicroscope at 35× magnification on high‐resolution epoxy casts. Microphotographs were captured using a Blackfly S digital camera with Kivy Mic Capture Z software. We used Helicon Focus 7 software to merge images from different focal planes to obtain a greater depth of field, and ImageJ to add scale bars. To minimize inter‐observer error, all specimens were analyzed by two independent experienced observers (IRP and FR). We included molars M1/m1, M2/m2 and, M3/m3, as outlined by Xafis et al. (2017). A 0.16 mm^2^ ocular reticle was used to quantify microwear features, including the number of pits (round scars), scratches (elongated, parallel‐side scars), gouges (irregular, and much larger and deeper than large pits), puncture pits (deep, crater‐like pits with low refractivity), cross scratches (perpendicular to most scratches), and scratch width score (SWS). An SWS score of “0” was given when only fine scratches were present, “1” when there was a mixture of fine and coarse scratches, “2” when coarse scratches were predominantly present, “3” when there was a mixture of coarse and hyper‐coarse scratches and “4” when the surface also had hyper‐coarse scratches. The mean numbers of scratches and pits were used to categorize dietary habits as browsers (eating woody and non‐woody dicotyledonous plants), grazers (eating grass), or mixed feeders by comparing the data to known dietary habits of extant ungulates and the three living elephant species (Solounias and Semprebon 2002). The bivariate microwear plots were generated in R (R Core Team 2023) using the MicrowearBivaR script (Rivals 2019).

For radiocarbon dating, enamel samples were processed at the Center for Applied Isotope Studies of the University of Georgia (United States) following standard laboratory procedures. Briefly, the enamel was ultrasonically cleaned, washed, dried, and gently crushed into small fragments. The fragments were treated with diluted 1 N acetic acid to remove surface and secondary carbonates, with periodic evacuation to remove evolved CO_2_ and ensure acid access to internal surfaces. The cleaned samples were then reacted under vacuum with 100% phosphoric acid to dissolve the bone mineral and release CO_2_ from bioapatite. The resultant CO_2_ was cryogenically purified and converted to graphite following Vogel et al. (1984). Graphite ^14^C/^13^C ratios were measured using AMS and compared with the Oxalic Acid I standard (NBS SRM 4990). Finally, uncalibrated radiocarbon ages are given in years BP (before 1950), based on a ^14^C half‐life of 5568 years, with 1*σ* errors reflecting both statistical and experimental uncertainties.

## 3 Results

### 3.1 Preservation of the Isotopic Signal

The samples analyzed in this study had an average ΔOCO3−PO418 value of approximately 9.3‰ (Supporting Information), aligning with the standard ΔOCO3−PO418 range for unaltered bioapatite of modern mammals (8.6‰–9.1‰) (Iacumin et al. 1996). This suggests the preservation of original $\delta^{18}O_{{\mathrm{CO}}_{3}}$ and $\delta^{18}O_{{\mathrm{PO}}_{4}}$ values. Additionally, the strong correlation between these isotopic values (*R* ^2^ = 0.96, *p* < 0.001) supports the interpretation that ${\mathrm{CO}}_{3}^{-2}$ and ${\mathrm{PO}}_{4}^{-3}$ components in bioapatite formed in isotopic equilibrium with body water under the relatively stable body temperatures characteristic of mammals.

### 3.2 Radiocarbon Dating

Radiocarbon dating results are summarized in Table 1. Dates were calibrated using Calib 8.0 (Stuiver and Reimer 2020) and the SHCal20 calibration curve (Hogg et al. 2020). Dates are reported as calibrated years Before Present (cal yr. BP).

All calibrated dates fall within the Late Pleistocene, with the oldest date obtained from Quebrada Colorada, Chimborazo (31,820 cal yr. BP) and the youngest from Pedro Pablo Gómez, Manabí (23,830 cal yr. BP). However, these ages should be interpreted cautiously because radiocarbon dates obtained from bioapatite can yield younger estimates than those derived from bone collagen and may therefore represent minimum ages.

### 3.3 δ^13^C Values

**FIGURE 2:**
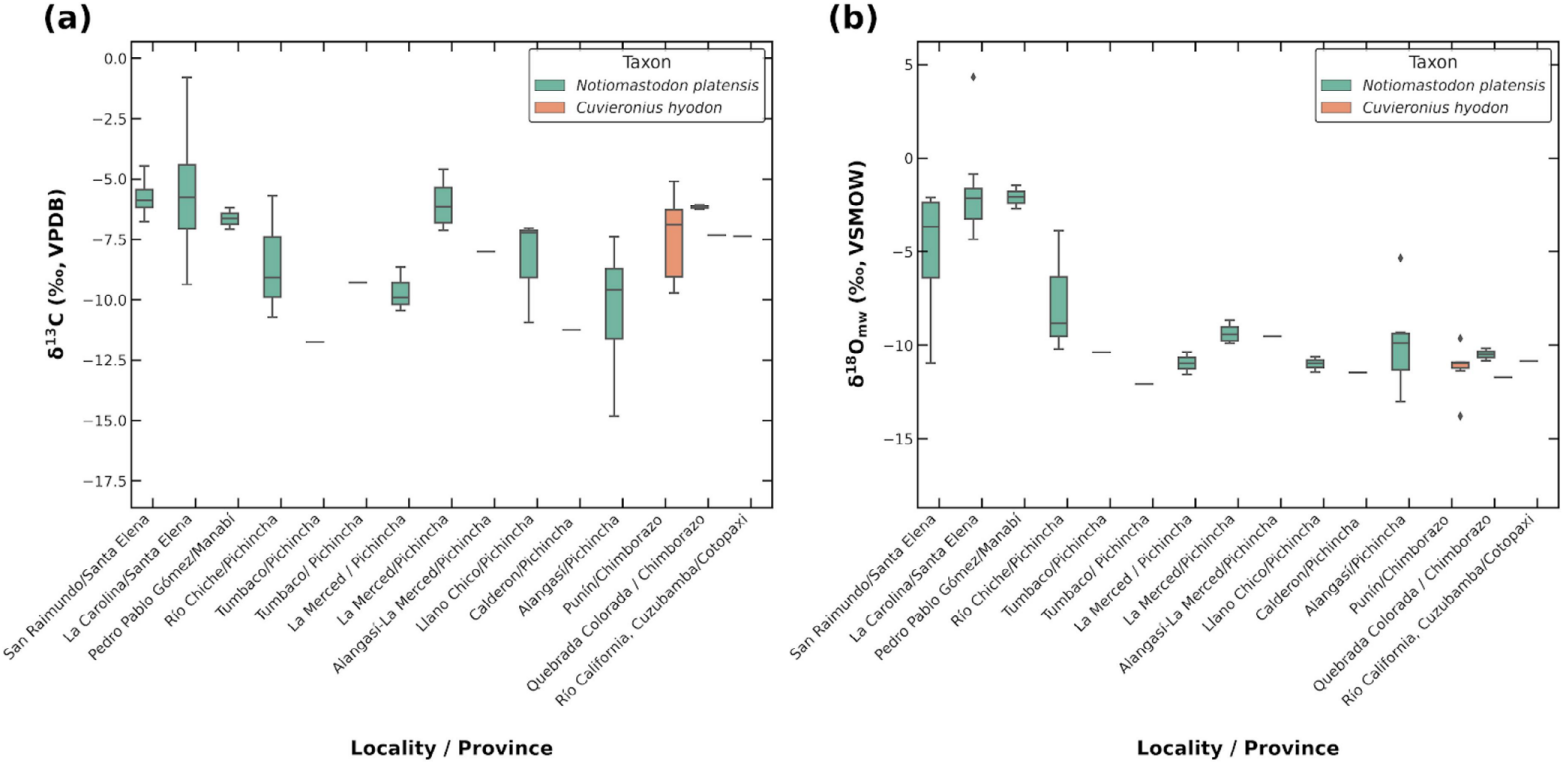
Box plots showing the carbon and oxygen isotopic composition of *Notiomastodon platensis* (green) and *Cuvieronius hyodon* (orange) across various localities and provinces of Ecuador. (a) δ^13^C values (‰, VPDB): Represent the carbon signatures obtained from tooth enamel, reflecting diet and the type of vegetation consumed (C_3_ vs. C_4_ plants). (b) δ^18^O_mw_ values (‰, VSMOW): Represent the isotopic composition of meteoric water reconstructed from the enamel values, indicating climatic variations and altitudinal effects in the hydrological basins.

The δ^13^C values for the entire sample ranged from −14.82‰ to −0.78‰. The average for *N. platensis* was −7.48‰ ± 2.6‰, while for *C. hyodon* it was −7.45‰ ± 1.8‰. A two‐way analysis of variance (ANOVA) revealed no significant differences in carbon isotope composition between taxa (*F*_1,40_ = 0.19, *p* = 0.665). However, a significant effect of locality was identified (*F*_12,40_ = 2.86, *p* = 0.006). Tukey's post hoc test confirmed that the greatest difference occurred between Alangasí, Pichincha (mean = −10.23‰), and La Carolina, Santa Elena (mean = −5.39‰), with a mean difference of 4.83‰ (*p* = 0.0014). Figure 2a shows a substantial overlap in the interquartile ranges (IQRs) between *N. platensis* and *C. hyodon* in the localities where they coexist, namely Punín and Alangasí. The medians of both taxa remain similar within each site, supporting the lack of dietary segregation between taxa. A greater amplitude is observed in the boxes of the lowland localities, particularly La Carolina, indicating greater variance in plant‐resource intake compared with the highland populations, whose boxes tend to be more compact and shifted toward more negative values.

### 3.4 Environmental Categories Inferred From δ^13^C Ranges

**FIGURE 3:**
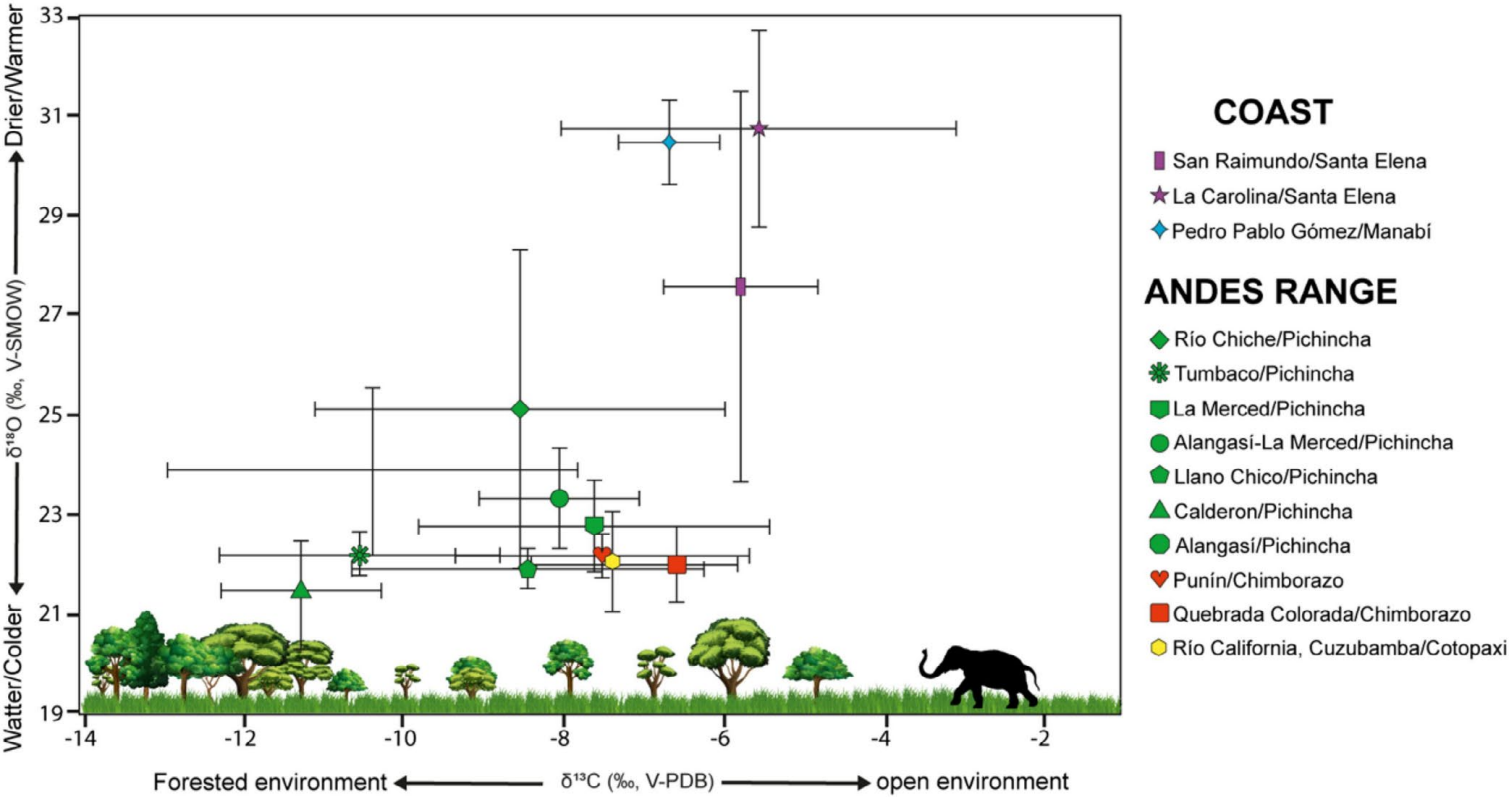
Mean enamel bioapatite δ^13^C (‰, VPDB) and δ^18^O_CO_3_ (‰, VSMOW) values ±1 SD from Ecuadorian gomphothere molars. The plot shows the inferred paleoenvironmental distribution of gomphotheres across Ecuadorian localities, based on stable isotope ranges established in previous studies (Domingo et al. 2012). The following paleoenvironments are represented: (i) mesic/woodland (−13.5‰ to −8.5‰), (ii) wooded C_3_ grassland to open and arid C_3_ grassland (−8.5‰ to −5.5‰), (iii) open C_3_–C_4_ vegetation (−5.5‰ to −0.5‰), and (iv) open C_4_ grassland (−0.5‰ to +7.5‰).

Based on δ^13^C ranges modified from Domingo et al. (2012), gomphothere specimens were assigned to the following paleoenvironmental categories by province: (1) In Santa Elena Province, δ^13^C values ranged from −9.39‰ to −0.78‰. Specimens showed δ^13^C values associated with mesic/woodland (*n* = 1), wooded C_3_ grasslands to open, arid C_3_ grasslands (*n* = 11), and open vegetation areas C_3_–C_4_ (*n* = 5). (2) In Manabí Province, gomphothere δ^13^C values ranged from −7.09‰ to −6.64‰, indicating wooded C_3_ grassland to open, arid C_3_ grassland (*n* = 2). (3) In Pichincha Province, δ^13^C values ranged from −14.82‰ to −4.58‰, indicating environments classified as closed canopy (*n* = 1), mesic/woodland (*n* = 13), wooded C_3_ grasslands to open, arid C_3_ grasslands (*n* = 9) and open vegetation areas C_3_–C_4_ (*n* = 1). (4) In Chimborazo Province, δ^13^C values ranged from −9.71‰ to −5.1‰, indicating mesic/woodland environments (*n* = 3), wooded C_3_ grasslands to open, arid C_3_ grasslands (*n* = 6) and open vegetation areas C_3_–C_4_ (*n* = 1). Samples from unknown localities ranged from −10.44‰ to −5.27‰, indicating environments ranging from mesic/woodlands (*n* = 3) to open vegetation areas C_3_–C_4_ (*n* = 1) (Figure 3).

### 3.5 Dietary Proportions and Niche‐Breadth Estimates

**FIGURE 4:**
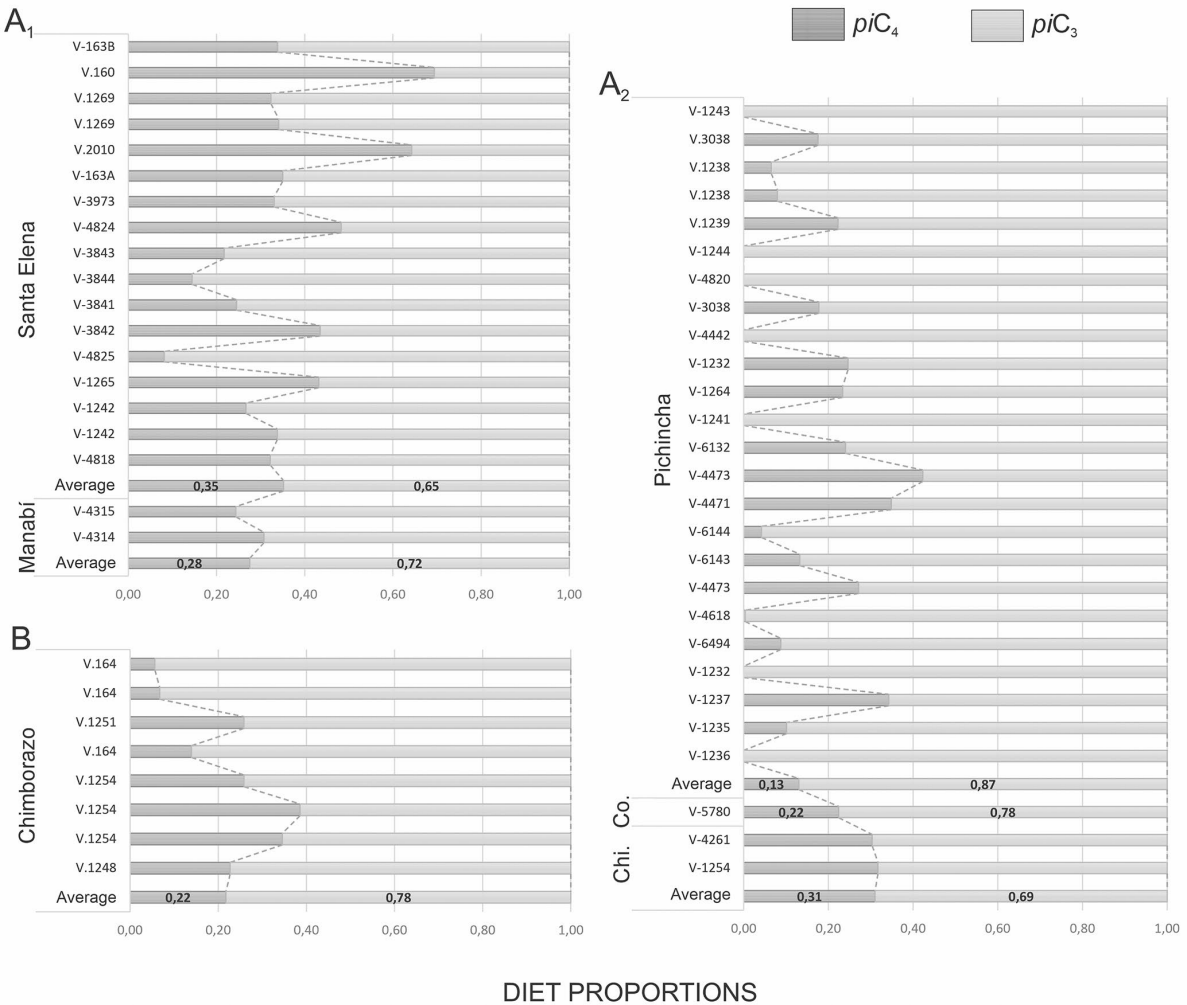
Estimated dietary proportions of C_3_ and C_4_ plant resources (p_iC_3_ and p_iC_4_) for Late Pleistocene gomphotheres from Ecuador. Panels A_1_ and A_2_ show dietary estimates for *Notiomastodon platensis*, and panel B shows dietary estimates for *Cuvieronius hyodon*.

The δ^13^C values of bioapatite samples from both gomphothere species in Ecuador are similar and indicate a broadly generalist feeding strategy, with a diet composed predominantly of C_3_ resources (mean, C_3_ = 77% and C_4_ = 23%, *B*_*A*_ = 0.51; Supporting Information and Figure 4), associated with habitats ranging from closed canopies to C_3_–C_4_ open landscapes (Figure 3). Differences between C_3_ and C_4_ plant consumption were mainly observed for *N. platensis* specimens from the coastal and Andean localities. In particular, specimens from Pichincha Province showed the lowest δ^13^C values (−9.02‰ ± 2.41‰), indicating substantial consumption of C_3_ plants in more forested landscapes. For these localities, some individuals may have had a specialized browser diet (C_3_ = 100%, *B*_*A*_ = 0), whereas others showed a mixed diet with higher proportions of C_3_ plants (means, *pi*C_3_ = 87% and *pi*C_4_ = 13%, *B*_*A*_ = 0.31; Supporting Information and Figure 4) in wooded and open C_3_–C_4_ grasslands. By contrast, *N. platensis* specimens from coastal localities, such as those from Santa Elena Province, showed higher δ^13^C (mean, −5.57‰ ± 2.18‰) associated with a diet composed of higher proportions of C_4_ plant resources in mixed C_3_/C_4_ habitats (means, *pi*C_3_ = 65% and *pi*C_4_ = 35%, *B*_*A*_ = 0.73; Supporting Information and Figure 4).

### 3.6 Altitudinal Gradient Inferred From δ^18^O Values

A statistical summary of the δ^18^O_meteoric water_ values calculated from gomphothere molars is provided in Table S4. The average for *N. platensis* was −7.08‰ ± 4.3‰ (range: −13.02‰ to 4.3‰), while that of *C. hyodon* was −11.1‰ ± 1.1‰ (range: −13.78‰ to −9.6‰). Permutational analysis of variance (PERMANOVA) revealed significant differences in oxygen isotope composition between taxa (Pseudo‐*F* = 7.72; *p* = 0.008), indicating differences in inferred water sources or altitudinal niches between *N. platensis* and *C. hyodon*. However, geographic provenance showed a substantially greater influence on the variability of the oxygen isotope signal (Pseudo‐*F* = 14.65; *p* = 0.001), indicating that local provenance was the main determinant of meteoric water isotopic signatures in the analyzed sample.

The boxplot shows a marked geographic segregation along the altitudinal gradient (Figure 2b). The medians for the Sierra localities, Pichincha and Chimborazo, are consistently below −8‰, whereas the coastal localities, represented by Santa Elena, have less negative medians, above −5‰. The dispersion of the data in *N. platensis* is notably greater than in *C. hyodon*, suggesting the use of more heterogeneous water sources or a broader altitudinal distribution. The outliers identified in San Raimundo and La Carolina represent individuals with signatures that deviate from the local trend, possibly linked to seasonal variation, local hydrological variability, or individual mobility.

**FIGURE 5:**
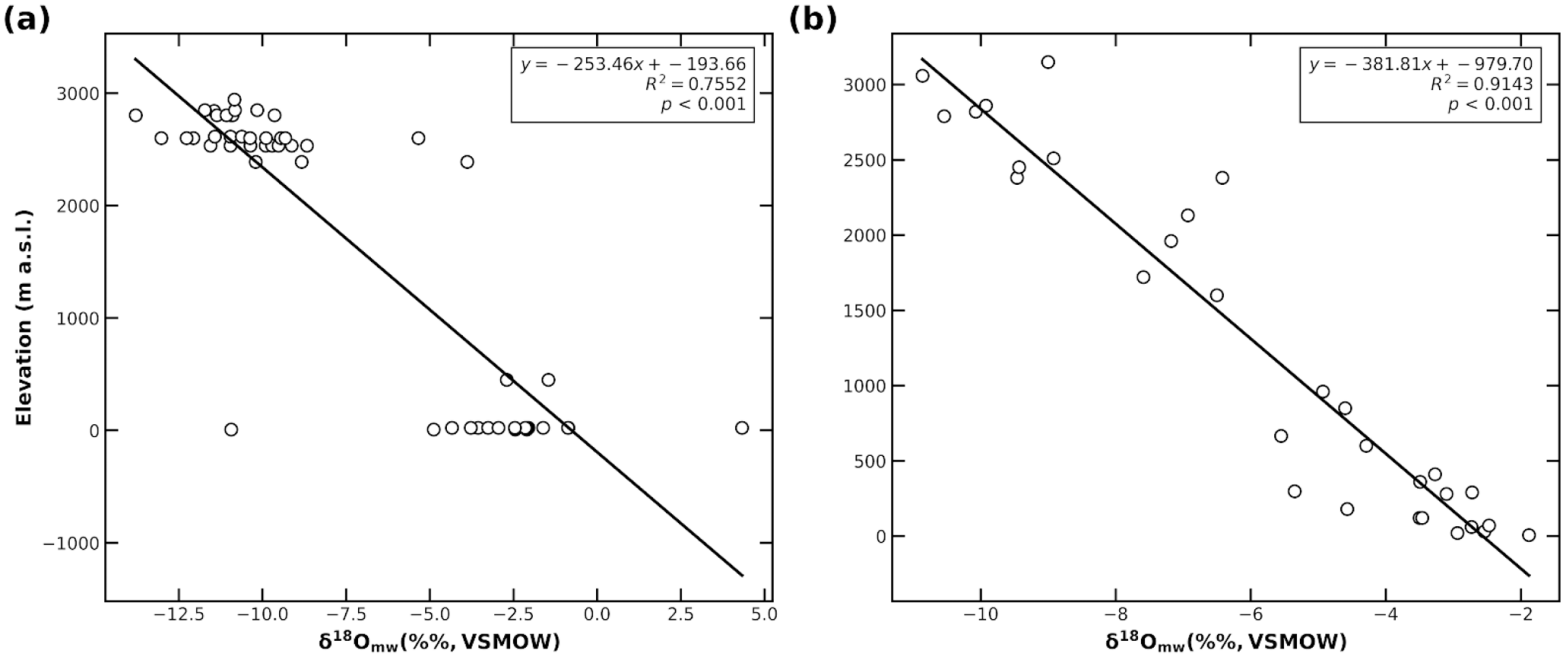
Linear regression between elevation and meteoric‐water δ^18^O values. (a) Relationship between reconstructed δ^18^O_mw values (‰, VSMOW) from gomphothere enamel samples and the elevation of fossil localities (m a.s.l.). (b) Relationship between modern meteoric‐water δ^18^O values (‰, VSMOW) and the elevation of meteorological stations (m a.s.l.). In both datasets, δ^18^O values decrease with increasing elevation, consistent with the expected altitudinal effect on meteoric‐water isotopic composition.

A strong negative association was observed between δ^18^O_meteoric water_ and altitude, suggesting decreasing δ^18^O values with increasing elevation, consistent with expected precipitation patterns (Rozanski and Araguás Araguás 1995) (*p* = 0.055, *ρ* = −0.752). The isotopic gradient of δ^18^O_meteoric water_ with altitude was calculated using a linear regression (*R* ^2^ = 0.755, *p* < 0.001), yielding a value of −0.30‰ per 100 m. A Kruskal–Wallis test was conducted to assess differences in δ^18^O values in modern water across localities. The results indicate no statistically significant differences between localities (*χ* ^2^ = 29, df = 29, *p* = 0.4651), suggesting similar δ^18^O distributions among sampling stations (Figure 5; Table S5).

### 3.7 Dental Microwear Analysis

**FIGURE 6:**
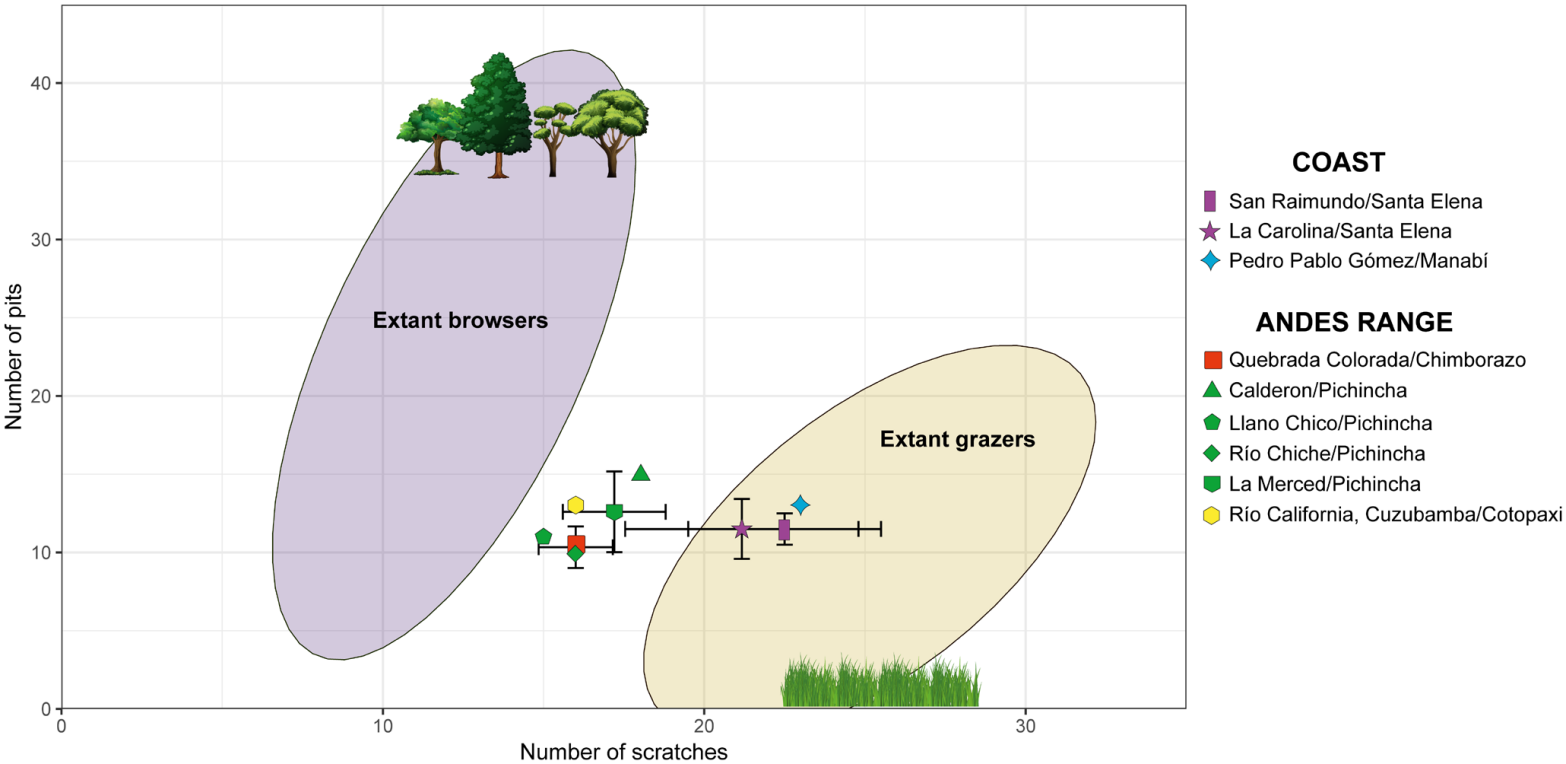
Bivariate graph showing the mean counts of pits and scratches in gomphotheres. Error bars represent one standard deviation (±1 SD) for the fossil specimens. The unfilled ellipses indicate the 95% Gaussian confidence intervals around the centroids of extant browsers and grazers, following the dataset compiled by Solounias and Semprebon (2002).

All Ecuadorian gomphotheres analyzed exhibit a consistent dietary signal, pointing to grazing or grass‐dominated mixed feeding (Figure 6). The 21 individuals examined display a higher number of scratches than pits (Table S2). When comparing the two geographic regions, coastal and Andean, the average number of pits is virtually identical in both regions: 11.7 in the coastal localities (Santa Elena and Manabí) and 11.9 in the Andean localities (Pichincha, Cotopaxi, and Chimborazo). Differences are more pronounced in the average number of scratches: 21.7 in the coastal localities (Santa Elena and Manabí) compared with 16.6 in the Andes (Pichincha, Cotopaxi, and Chimborazo). This contrast suggests that gomphotheres in the coastal lowlands consumed a more abrasive diet. All specimens exhibit hyper‐coarse scratches, large pits, cross scratches, gouges, and puncture pits, with the exception of specimen V‐6130 (Table S2).

## 4 Discussion

### 4.1 Chronology of the Samples Analyzed

All individuals analyzed derive from Late Pleistocene deposits of the Tablazo, Jama, and Cangahua formations (see Supporting Information). However, most of the specimens analyzed in this study lack precise stratigraphic, depositional, and chronological control. Considering the scarcity of radiocarbon dates for extinct megafauna from Ecuador, and acknowledging the limitations of our dating approach, the new dates presented here (Table 1) show general agreement with previously published results.

This consistency is evident, for example, at the site of Tanque Loma in the coastal region, where Lindsey and López (2015) reported three radiocarbon dates obtained from bone collagen. Two of these correspond to different specimens of *N. platensis* and fall within the Late Pleistocene: 20,750 cal yr. BP (CAMS160800: 17,170 ± 920 ^14^C yr. BP) and 23,010 cal yr. BP (CAMS160801: 19,110 ± 1260 ^14^C yr. BP). The third date, corresponding to the giant ground sloth *Eremotherium laurillardi*, is older (27,680 cal yr. BP; CAMS‐147211: 23,560 ± 180 ^14^C yr. BP).

Additional radiocarbon dates have been reported for the northern Andean region, specifically from Quebrada Cuesaca and Quebrada Pistud (Coltorti et al. 1998). Although that study does not provide lab numbers, dating method, or material dated beyond a general reference to “bone”, it reports one date for *N. platensis* (20,100 cal yr. BP; ^14^C age: 16,670 ± 80 yr. BP) and another for *Glossotherium wegneri* (14,330 cal yr. BP; ^14^C age: 12,350 ± 70 yr. BP). Although both dates are younger than those reported here, they still fall within the Late Pleistocene.

Finally, although conversion methods have been proposed to convert bioapatite radiocarbon dates to collagen‐equivalent ages (Dantas and Cherkinsky 2023), we opted to report bioapatite dates without applying such conversions. This decision reflects the wide range of environmental settings considered in this study, as well as the fact that existing conversion models are based on relatively limited datasets and are calibrated for specific environmental conditions.

### 4.2 Paleoenvironmental Pattern

**FIGURE 7:**
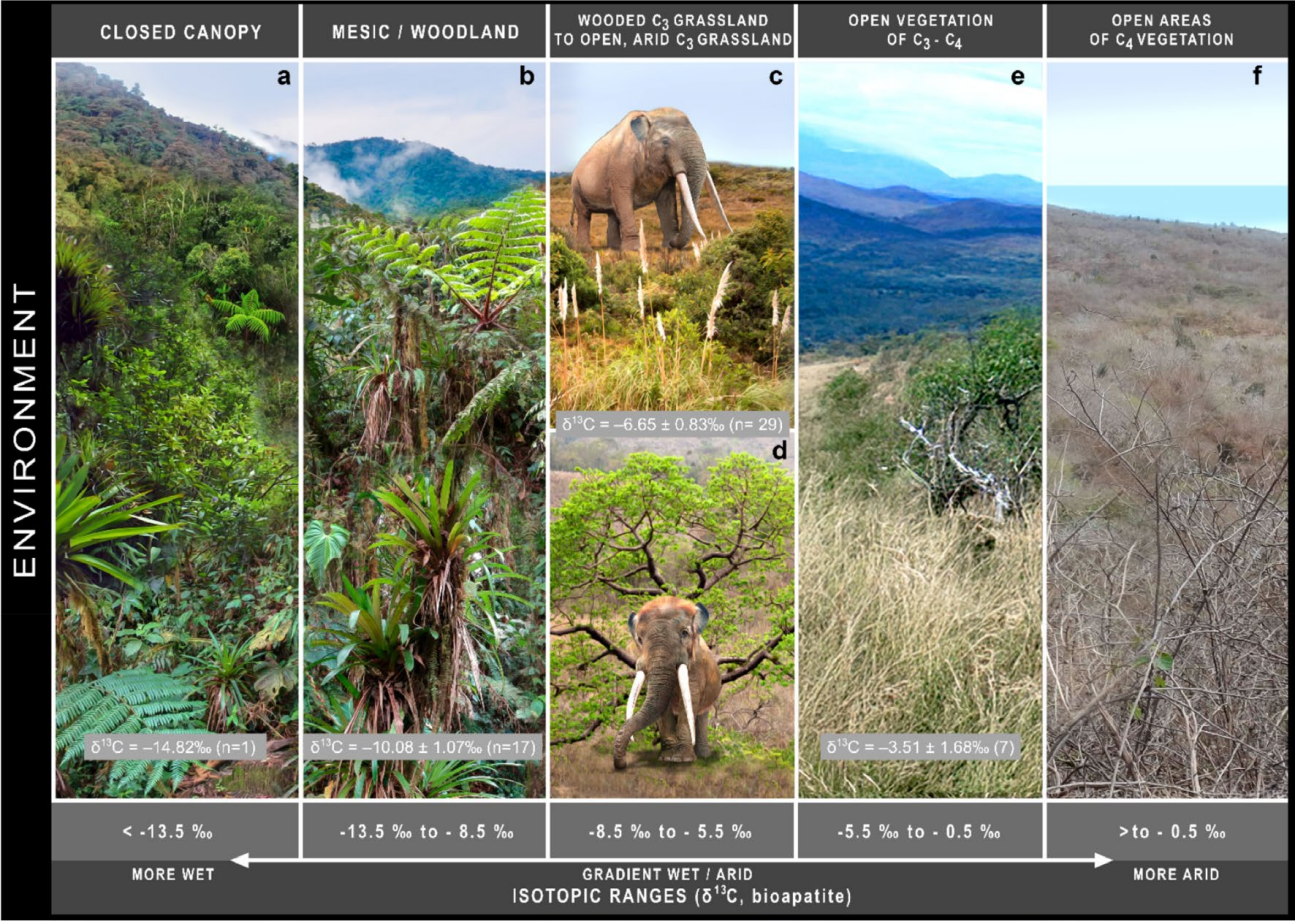
Isotopic range of enamel bioapatite δ^13^C values (‰, VPDB) in the study area, corresponding to different vegetation types. Most of the analyzed specimens lived in environments ranging from wooded C_3_ grassland to open arid C_3_ grassland. Vegetation types are indicated as follows: (a) Western Montane Forest, (b) More open Western Montane Forest, (c) Inter‐Andean scrubland, (d) Coastal Deciduous Forest, (e and f) Dry Coastal Scrubland.

Regarding vegetation cover, the environmental ranges established by stable isotope analysis indicate that gomphotheres in Ecuador inhabited a wide variety of environments as reflected by enamel bioapatite δ^13^C values ranging from −14.82‰ to −0.78‰. However, there was a trend toward the use of relatively open and mixed environments, including grasslands, shrublands, and wooded grasslands (Figure 3). The mean value of the entire dataset indicates wooded C_3_ grassland to open, arid C_3_ grassland environments (δ^13^C = −7.55‰ ± 2.5‰; Figure 7). These results differ from those reported for gomphotheres from the Southern Cone of South America, where Chilean gomphotheres appear to have relied more heavily on forest resources (González‐Guarda et al. 2025). After applying the diet–bioapatite enrichment factor ($\varepsilon_{\text{diet}-\text{bioapatite}}^{*}$), the estimated consumed‐diet δ^13^C values also support the presence of relatively open and/or dry vegetation cover in the environments occupied by Ecuadorian gomphotheres (δ^13^C_Estimated Consumed Diet_ = −22.55‰ ± 2.5‰; see Kohn 2010). Regarding the photosynthetic pathways of consumed plants, some specimens show values consistent with diets dominated by C_3_ vegetation, whereas others indicate mixed C_3_–C_4_ resource consumption.

The analysis of dental microwear showed a predominance of scratches (Figure 6; Table S2) particularly among specimens from coastal localities (e.g., Santa Elena Province), corroborating the results obtained from δ^13^C. Thus, grass consumption constituted an important component of the diet of these gomphotheres; however, a large portion of individuals also fed on C_3_ vegetation, including both grasses more prevalent at high elevations and C_3_ trees and shrubs. Therefore, as in extant elephants, seasonal variation may have influenced the dietary composition of these individuals (Baskaran and Desai 2013; Beirne et al. 2021).

### 4.3 Environmental Variability

Environmental variability is particularly evident in Pichincha Province. Here, the dataset includes the only individual with a δ^13^C value consistent with closed‐canopy forest conditions (δ^13^C = −14.82‰; Alangasí/Pichincha; 2600 m a.s.l). Today, the Alangasi site is characterized by Andean shrub vegetation. More broadly, Pichincha Province contains elements of montane forest, Andean scrub, and páramo. Therefore, the isotopic signature suggests that this individual inhabited the area during an interval of montane forest expansion, probably related to an increase in temperature and precipitation in the area at the time. Unfortunately, no date is available for this individual. One possibility is that this individual lived during the Last Glacial Termination, as the pattern of forest expansion at high altitudes began around 16,000 years ago in Ecuador (Heusser and Shackleton 1994). However, this does not mean that there were no brief (e.g., interstadial) intervals of forest expansion during the Last Glacial Maximum, nor areas of climatic microrefuge (Ziegler et al. 2025; preprint). In fact, the expansion of high‐mountain forests such as *Podocarpus* has been documented between 34,000 and 28,000 years BP (Heusser and Shackleton 1994).

A second group of individuals shows values consistent with a mesic woodland environment (δ^13^C = −10.65‰ ± 1.6‰) (*n* = 14), indicating a greater contribution of arboreal C_3_ resources and possibly more humid climatic conditions (Kohn 2010). The other environmental signal recorded in Pichincha Province ranges from wooded C_3_ grassland to open, arid C_3_ grassland (−6.74‰ ± 2.2‰) (*n* = 10), a pattern more compatible with the present‐day vegetation of Pichincha. For example, sample V‐6143, radiocarbon dated between 25,344 and 25,003 cal yr. BP from the locality of La Merced, adjacent to Alangasi, exhibits the paleoenvironmental variability expected in Pichincha Province during the Late Pleistocene; this specimen falls close to the isotopic threshold (δ^13^C = −8.65‰) between wooded C_3_ grassland to open, arid C_3_ grassland and mesic/woodland conditions. Regarding specimens from inland locations (Pichincha, Chimborazo, and Cotopaxi), dental microwear data corroborate the interpretation of a mixed diet in agreement with the isotopic results. However, the specimens from Pichincha Province, which had more negative δ^13^C values, appear to have included a greater proportion of browse or arboreal resources in their diet. Notably, the isotopic and microwear signal of gomphotheres from Pichincha Province resembles the mixed‐feeding behavior interpreted for *N. platensis* from Tibitó, a locality in the Inter‐Andean Valley of Colombia (2565 m a.s.l.) at an elevation comparable to Pichincha. The results from Tibitó suggest that these gomphotheres (*n* = 4) were capable of exploiting different types of vegetation according to seasonally varying availability (Ziegler et al. 2025; preprint).

### 4.4 Paleoclimate and Gomphothere Mobility

The high values of δ^18^O_meteoric water_ (*n* = 19; −2.6‰ ± 2.7‰) obtained from gomphotheres from the coastal area could indicate evaporated water sources under relatively warm and/or arid tropical conditions. This pattern holds with the exception of sample V‐1242 from San Raimundo (Santa Elena Province), which shows a much more negative value (δ^18^O_meteoric water_ = −10.94‰) than the other samples from the coastal area. Two non‐mutually exclusive explanations may account for this difference. First, the individual may have primarily consumed water from rivers that originated directly from a nearby mountain, such as the Chongón‐Colonche range and associated drainage network. River water originating in the mountains generally has more negative δ^18^O_meteoric water_ values (Taucare et al. 2020), even in low‐altitude areas and near the mouth of the same river (González‐Guarda, E., pers. comm., 2026). Thus, under severe drought conditions, when lentic waters such as wetlands and lagoons had completely dried up, river water could have been the only available drinking‐water source. Second, this individual may have consumed precipitation‐derived water with significantly depleted δ^18^O values, consistent with the effects of convective storms over the Pacific Ocean on δ^18^O values along the Ecuadorian coast (Garcia et al. 1998). Future work based on new isotopic baselines (e.g., baseline of δ^18^O_meteoric water_ from different hydrological sources from the sea to the Andes) would help better understand the results obtained for this particular sample.

In the coastal area and Inter‐Andean valleys, evidence of arid conditions and grassland environments is supported by dental microwear. This interpretation is consistent with the δ^13^C values indicating a trend toward more open environments (δ^13^C = −5.68‰ ± 2‰), although we suggest that most gomphotheres included leaves and branches in their diet as well. In addition, the presence of C_4_ vegetation in some individuals, suggests aridity. However, caution should be exercised when interpreting results from fossil findings above 3000 m a.s.l., as these elevations correspond to páramo ecosystems characterized by high humidity and open environments (Frederick et al. 2018). Tree biomass in this ecosystem is limited, in part due to edaphic and climatic constraints. Sample V‐4825 (δ^13^C = −9.36‰), from La Carolina, Santa Elena Province is an exception, suggesting a more humid environment with the presence of arboreal elements.

The two dated specimens from coastal sites, La Carolina (Santa Elena Province) and Pedro Pablo Gómez (Manabí Province), have calibrated age ranges of 24,868–24,305 and 23,946–23,749 cal yr. BP (2 sigma), respectively. Both samples reflect the altitudinal effect, consistent with the modern isotopic trend (Supporting Information; Figure 5). However, given the high δ^18^O_meteoric water_ values, these gomphotheres would have been drinking water from highly evaporated hydrological sources (δ^18^O_meteoric water_ = −0.86‰; −1.46‰), probably from stagnant waters like wetlands. In addition, the δ^13^C values of the dated samples follow the open environment pattern for coastal sites (δ^13^C = −3.7‰; −6.1‰). Particularly, the δ^13^C value of specimen V‐4824 (La Carolina site; δ^13^C = −3.7‰; cal mean = 24,606 cal yr. BP) suggests that, during the LGM, favorable climatic conditions existed for C_4_ plants to be locally available.

Interestingly, at higher altitude, evidence of C_4_ plant environments was recorded at the La Merced site (Pichincha; δ^13^C = −4.58‰; 2534 m a.s.l) and at the Punín site (Chimborazo, δ^13^C = −5.1‰; 2803 m a.s.l). A C_4_ signal has also been recorded in gomphotheres from Tarija in Bolivia (1866 m a.s.l; Sánchez et al. 2004) and Ayusbamba in Peru (3783 m a.s.l; Domingo et al. 2012, 2020). Our evidence is consistent with paleoenvironmental pollen records from the Colombian páramo during the Last Glacial Maximum. At that time, C_4_ vegetation expanded in the páramo up to elevations of approximately 3500 m, whereas today, C_4_‐dominated vegetation occurs below 2200 m a.s.l. (Hooghiemstra and Van der Hammen 2004). Ziegler et al. (2025; preprint) document C_4_ vegetation consumption by *N. platensis* at 1626 m a.s.l. (Anolaima locality). During the LGM, low atmospheric CO_2_ partial pressure (pCO_2_) may have favored the greater abundance of C_4_ plants in páramo ecosystems (Boom et al. 2001).

Dated samples from higher altitude locations show semi‐open and arid environments (δ^13^C = −7.35‰ ± 1.29‰), and colder environments (δ^18^O_meteoric water_ = −10.65‰ ± 0.43‰). Despite the wide temporal range between the three dated samples (32,066–25,003 cal yr. BP; Table 1), we did not observe significant variations. Interestingly, the δ^18^O_meteoric water_ values calculated from gomphothere tooth enamel are similar to modern δ^18^O_meteoric water_ values (Supporting Information), both in coastal and Andean areas. The δ^18^O values of meteoric water recorded by Andean gomphotheres do not show a clear signal of strong influence from Atlantic‐derived air masses, as these air masses reach the Inter‐Andean depression enriched in ^18^O after crossing the Amazon Basin, due to high evapotranspiration (Salati et al. 1979). Furthermore, both in the gomphotheres and in the modern values, it can be observed that the δ^18^O_meteoric water_ values are affected by the altitudinal gradient (Figure 5; isotopic gradient: −0.30‰ per 100 m for gomphotheres; −0.25‰ per 100 m for modern meteoric water), which is consistent with recent paleoaltimetry studies (i.e., Gébelin et al. 2021; −0.15‰ per 100 m). Taken together, these preliminary results suggest that meteoric‐water δ^18^O values related to atmospheric circulation in Ecuador have not undergone major changes from the Late Pleistocene to the present.

In the dry Santa Elena Peninsula, on the Ecuadorian coast, abundant water bodies have been hypothesized during the Late Pleistocene, based on the composition of avian assemblages documented in the region (Campbell 2010). Accordingly, palaeoecological niche modeling of extinct megafauna suggests that these large mammals were able to persist in both the Santa Elena Peninsula and the Inter‐Andean Valley during the late Pleistocene and into the middle Holocene (6 kya; Araújo et al. 2021). In other areas, the adjacent Andean foothills may have supported premontane forests because they were unable to migrate into the arid lowlands. A recent phylogeographic study suggests that the Andean foothills of southwestern Ecuador may have provided suitable environmental conditions for the presence of humid forests during the Pleistocene (Escobar et al. 2021). However, paleoenvironmental evidence from the gomphotheres analyzed in this study, together with pollen records (Heusser and Shackleton 1994; Hansen et al. 2003), and phylogeographic data (Escobar et al. 2021), indicate that the lowlands of southwestern Ecuador remained dry during recent glacial cycles. Therefore, given that the δ^13^C isotopic signature is an average of the dental enamel growth time (approx. 3 years in proboscideans), part of their diet may have consisted of woody vegetation during the driest season, either from the consumption of leaves of trees adapted to aridity or during seasonal movements toward vegetation patches located in valleys.

However, the molars (*n* = 9) of gomphotheres from Colombia, a country with environments and climates similar to those of Ecuador, do not show substantial changes in their habitat (e.g., from grasslands to woodlands in the same individual) when part of the life history of each individual, approximately 3 years, was evaluated through sequential δ^13^C sampling (Ziegler et al. 2025; preprint). A similar pattern is observed for δ^18^O values, although one individual shows greater dispersion (sample TBT‐08: −6.22‰ ± 1.8‰), which also exhibits the highest dispersion among δ^13^C values (−11.97‰ ± 1.7‰). In any case, this variation occurs within a context of very similar environments, ranging from dense forest to mesic/woodland. Consequently, in the Colombia sample, there is variation between individuals from different locations (from dense forest to C_4_ grasslands), but no clear evidence for substantial intra‐individual variation.

### 4.5 Refuge Theory

A recent phylogeographic and paleoclimatic study of the endemic palm *Phytelephas aequatorialis* (Escobar et al. 2021) suggests that populations inhabiting the Andean foothills of northwestern Ecuador migrated downslope into the lowlands during glacial cycles, consistent with the Moist Forest Model, while maintaining connectivity with populations in the Chocó refugium. In contrast, southern populations may have taken refuge in a narrow strip of Andean foothills in southwestern Ecuador. These populations could have survived the Pleistocene in isolation on the Andean slopes, as they were unable to migrate to the lowlands of southwestern Ecuador due to the aridity of these areas during glacial cycles. Unlike the northern populations, the southern ones are consistent with the DRM.

In our study, the multiproxy approach also supports the DRM for the following reasons:

1. In the coastal region (Santa Elena and Manabí Provinces), the isotopic and dental microwear evidence indicates predominantly open and relatively arid environments. At present, these environmental conditions are favored by strong aridity forcing mechanisms, including the cold Humboldt Current, which inhibits the formation of rain clouds, subtropical anticyclones that generate dry air masses, and the Andes Mountain range, which blocks humid easterly winds.
2. At mid to high elevations, in the Inter‐Andean Valley, our results indicate a trend toward drier and more open environments. Today, such conditions are widespread in this region and result from the orographic effects of the western and eastern Andes Range that act as natural barriers to moisture. Humid air masses from both the Pacific Ocean and the Amazon Basin are forced to rise along the outer slopes of the Andes, where cooling and condensation produce significant precipitation in the foothills and at higher elevations, allowing the existence of cloud forests and humid páramos. Once the air passes over the peaks and descends into the interior of the Andean Valley, it has lost most of its moisture (Montilla‐Pacheco et al. 2024). Nevertheless, wetter and forested patches persist in the region, suggesting environmental heterogeneity. During many periods of the Pleistocene, the valley may have resembled this current configuration (Figure 1), consistent with our results indicating a more heterogeneous environment at the time of the gomphotheres' occupation of these landscapes.
3. In addition, at mid‐ to high elevations, isotopic evidence of C_4_ vegetation consumption by some individuals further supports the presence of open and dry environments.

Finally, while our research cannot rule out alternative interpretations that challenge the DRM hypothesis (Hostetler and Mix 1999; Colinvaux and De Oliveira 2001) the weight of evidence from our study supports this framework. Given the compelling evidence documenting generalist/opportunistic behavior in gomphotheres (Asevedo et al. 2020), a greater signal for forested environments in Ecuador would be expected if such habitats had been widely present during the Late Pleistocene. However, this pattern is not observed in our results.

Our findings in the inter‐Andean Valleys of Ecuador are consistent with the recent proposal by Ziegler et al. (2025; preprint), who suggest that the northern Andean highlands may have acted as stable refuges for megafauna owing to habitat diversity and a comparatively reduced impact of glacial environmental change. Notwithstanding the above, future work should also evaluate whether the arid lowlands of Ecuador were not also “ecological buffers”, considering that megafauna inhabited the Provinces of Santa Elena and Manabí for thousands of years. Further studies with empirical evidence are still needed to determine how climatic and environmental instability, if it occurred, in other regions of South America may have actually affected the decline in gomphothere populations.

## 5 Conclusions

Enamel bioapatite δ^13^C values and dental microwear data indicate that gomphotheres from Ecuador lived in diverse environments, but mostly in open to semi‐open and relatively arid habitats. In the coastal area, the δ^18^O signal of meteoric water indicates a predominantly warm climate, while at higher elevations the climate was colder.

From a paleoclimatic perspective, comparisons between δ^18^O meteoric water values from gomphotheres and modern sources in Ecuador show no major differences. Therefore, it is possible that the climatic fluctuations of the Late Pleistocene did not substantially modify the δ^18^O meteoric water values, at least within the chronological range represented by the sampled individuals. This pattern suggests relative stability in the isotopic composition of meteoric water and in the main atmospheric circulation controls affecting Ecuador from the Late Pleistocene to the present.

From a paleoecological perspective, our study contributes to a better understanding of paleobiogeographic patterns of Ecuadorian vegetation and provides new insights into the paleodiet of extinct herbivorous mammals. First, our results support the DRM, primarily in the Inter‐Andean Valley where forests may have been restricted to mid‐elevations due to their proximity to adjacent dry areas. In contrast, our results from the coastal area support the DRM only indirectly, since they do not provide evidence for the persistence of vegetation in the mountains, but rather demonstrate that this part of the Ecuadorian coast was dry during the Pleistocene, similar to present‐day conditions. Second, we present new evidence supporting the idea that the diet of generalist/opportunistic herbivorous mammals appears to be more limited by environmental resource availability than by the dietary breadth estimated through morpho‐functional inferences (e.g., molars adapted for cutting, crushing, grinding, etc.).

Consequently, the gomphothere populations of Ecuador and Chile constitute contrasting case studies in South American paleoecology, exhibiting different dietary responses to the diverse environments they inhabited.

## Author Contributions

**Erwin González‐Guarda:** conceptualization (equal), data curation (equal), formal analysis (equal), funding acquisition (equal), investigation (equal), writing – original draft (equal), writing – review and editing (equal). **Iván Ramírez‐Pedraza:** conceptualization (equal), formal analysis (equal), investigation (equal), writing – review and editing (equal). **Carlos Tornero:** investigation (equal), resources (equal). **Lidiane Asevedo:** investigation (equal). **Natalia Villavicencio:** investigation (equal). **Sebastián Escobar:** investigation (equal). **José Luis Román:** investigation (equal). **Melissa Hidalgo:** investigation (equal). **Esteban Benalcázar:** investigation (equal). **Florent Rivals:** investigation (equal), resources (equal).

## Funding

This study was supported by Fondo Nacional de Desarrollo Científico y Tecnológico (11241036) and by the READ project, funded by the European Research Council (ERC) under the European Union's HORIZON ERC programme (Grant agreement No. 101088071).

## Conflicts of Interest

The authors declare no conflicts of interest.

## Supporting information


**Appendix S1:** Description of the current climate and vegetation of the study area, paleoenvironmental background, principles of stable isotope and dental microwear analyses, and assessment of the preservation of the original isotopic composition.

**Data S1:** (1) Specimen‐level contextual, chronological, and stable isotope data for Late Pleistocene gomphotheres from Ecuador, including locality, taxonomy, specimen information, geographic coordinates, altitude, δ¹³C values, estimated dietary δ¹³C values, δ¹⁸O values of enamel carbonate and phosphate, reconstructed meteoric‐water δ¹⁸O values, carbonate–phosphate offsets used to assess diagenetic preservation, and data sources. (2) Standardized δ¹³C and δ¹⁸O values, estimated proportional contributions of C₃ and C₄ plants to the diet (piC₃ and piC₄), and standardized isotopic niche breadth (B_A). (3) Summary statistics for modern meteoric‐water δ¹⁸O values from Ecuadorian monitoring stations, including altitude, number of samples (*n*), maximum, minimum, mean, and SD.

**Table S1:** Summary of stable isotope data (δ^13^C, ‰V‐PDB) of the molar specimens from the gomphotheres of Ecuador. Number of samples (*n*), maximum (Max), minimum (Min), mean values and standard deviation (SD).

**Table S2:** Dental microwear data of gomphothere specimens from the Ecuador. Abbreviations: *n*, number of specimens; NS, average number of scratches; NP, average number of pits; SWS, scratch width score (0 = only fine scratches, 1 = mixture of fine and coarse scratches, 2 = predominant coarse scratches, 3 = mixture of coarse and hypercoarse scratches, 4 = presence of hypercoarse scratches); PP, presence or absence (0/1) of puncture pits; HC, presence or absence (0/1) of hyper‐coarse scratches; LP, presence or absence (0/1) of large pits; G, presence or absence (0/1) of gouges, and XS, presence or absence (0/1) of cross scratches.

**Table S3:** Summary of stable isotope data (δ^13^C, ‰V‐PDB) and the Estimated Consumed Diet of the gomphotheres specimens from the Ecuador. Number of samples (*n*), maximum (Max), minimum (Min), mean values and standard deviation (SD).

**Table S4:** Summary data of δ^13^C_VPDB_, proportional contributions of diet sources (*pi*C_3_ and *pi*C_4_ plants) and standardized isotopic niche breadth (*B*_*A*_) of the Pleistocene gomphotheres from the Ecuador. Number of samples (*n*), maximum (Max), minimum (Min) and Mean values.

**Table S5:** Statistical summary of stable isotope data (δ^18^O_meteoric water_, ‰V‐SMOW) of the gomphothere specimens from the Ecuador. Number of samples (*n*), maximum (Max), minimum (Min), mean values and standard deviation (SD).

## Data Availability

The DAS describes the availability of data. The data for this article are available in an Excel file called Supporting Information.

## References

[ece374099-bib-0001] Araújo, T. , H. Machado , D. Mothé , and L. dos Santos Avilla . 2021. “Species Distribution Modeling Reveals the Ecological Niche of Extinct Megafauna From South America.” Quaternary Research 104: 151–158. 10.1017/qua.2021.24.

[ece374099-bib-0002] Asevedo, L. , C. D'Apolito , S. Y. Misumi , M. A. Barros , O. M. Barth , and L. S. Avilla . 2020. “Palynological Analysis of Dental Calculus From Pleistocene Proboscideans of Southern Brazil: A New Approach for Paleodiet and Paleoenvironmental Reconstructions.” Palaeogeography Palaeoclimatology Palaeoecology 540: 109523.

[ece374099-bib-0003] Asevedo, L. , T. R. Pansani , V. M. Cordeiro , et al. 2021. “Diversity of Pleistocene Megamammals From Southern Amazon, Mato Grosso State, Brazil.” Journal of South American Earth Sciences 112: 103552. 10.1016/j.jsames.2021.103552.

[ece374099-bib-0004] Ayliffe, L. K. , A. M. Lister , and A. R. Chivas . 1992. “The Preservation of Glacial‐Interglacial Climatic Signatures in the Oxygen Isotopes of Elephant Skeletal Phosphate.” Palaeogeography Palaeoclimatology Palaeoecology 99: 179–191.

[ece374099-bib-0005] Baskaran, N. , and A. A. Desai . 2013. “Frugivory and Seed Dispersal by the Asian Elephant *Elephas maximus* in the Tropical Forests of Nilgiri Biosphere Reserve, Southern India.” Journal of Threatened Taxa 5: 4893–4897. 10.11609/JoTT.o2848.4893-7.

[ece374099-bib-0006] Beerling, D. J. , and F. E. Mayle . 2006. “Contrasting Effects of Climate and CO_2_ on Amazonian Ecosystems Since the Last Glacial Maximum.” Global Change Biology 12, no. 10: 1977–1984.

[ece374099-bib-0007] Beirne, C. , T. M. Houslay , P. Morkel , et al. 2021. “African Forest Elephant Movements Depend on Time Scale and Individual Behavior.” Scientific Reports 11: 12634. 10.1038/s41598-021-91627-z. 34135350 PMC8208977

[ece374099-bib-0008] Benalcázar, E. 2025. “Biomecánica Mandibular en Proboscídea: Estudio Comparativo con Enfoque en *Notiomastodon platensis*.” Tesis de magíster. Facultad de Ciencias, Universidad Austral de Chile, Valdivia, Chile, 106 p.

[ece374099-bib-0009] Boom, A. , G. Mora , A. M. Cleef , and H. Hooghiemstra . 2001. “High Altitude C_4_ Grasslands in the Northern Andes: Relicts From Glacial Conditions?” Review of Palaeobotany and Palynology 115, no. 3–4: 147–160. 11440767 10.1016/s0034-6667(01)00056-2

[ece374099-bib-0010] Campbell, K. E. 2010. “Global Climate Dynamics and Plio‐Pleistocene Paleoenvironments of Northwestern Peru and Southwestern Ecuador.” In: XV Congreso Peruano de Geología, Resúmenes Extendidos. Sociedad Geológica del Perú, Publicación Especial Nº 9, 424–427.

[ece374099-bib-0011] Carnaval, A. C. , M. J. Hickerson , C. F. B. Haddad , M. T. Rodrigues , and C. Moritz . 2009. “Stability Predicts Genetic Diversity in the Brazilian Atlantic Forest Hotspot.” Science 323: 785–789. 10.1126/science.1166955. 19197066

[ece374099-bib-0012] Cheng, H. , A. Sinha , F. W. Cruz , et al. 2013. “Climate Change Patterns in Amazonia and Biodiversity.” Nature Communications 4: 1411. 10.1038/ncomms2415. 23361002

[ece374099-bib-0013] Colinvaux, P. A. , M. B. Bush , M. Steinitz‐Kannan , and M. C. Miller . 1997. “Glacial and Postglacial Pollen Records From the Ecuadorian Andes and Amazon.” Quaternary Research 48: 69–78. 10.1006/qres.1997.1908.

[ece374099-bib-0014] Colinvaux, P. A. , and P. E. De Oliveira . 2001. “Amazon Plant Diversity and Climate Through the Cenozoic.” Palaeogeography Palaeoclimatology Palaeoecology 166: 51–63. 10.1016/S0031-0182(00)00201-7.

[ece374099-bib-0015] Coltorti, M. , G. Ficcarelli , H. Jahren , M. Moreno Espinosa , L. Rook , and D. Torre . 1998. “The Last Occurrence of Pleistocene Megafauna in the Ecuadorian Andes.” Journal of South American Earth Sciences 11, no. 6: 581–586. 10.1016/S0895-9811(98)00037-6.

[ece374099-bib-0016] Dantas, M. A. T. , and A. Cherkinsky . 2023. “Interrelation of Radiocarbon Ages From Bone Fractions in the Brazilian Intertropical Region.” Quaternary Research 115: 202–206. 10.1017/qua.2023.19.

[ece374099-bib-0017] Dantas, M. A. T. , A. Cherkinsky , C. M. B. Lessa , et al. 2020. “Isotopic Paleoecology (δ^13^C, δ^18^O) of Late Quaternary Megafauna From the Brazilian Intertropical Region.” Revista Brasileira de Paleontologia 23: 138–152.

[ece374099-bib-0018] Domingo, L. , J. L. Prado , and M. T. Alberdi . 2012. “The Effect of Paleoecology and Paleobiogeography on Stable Isotopes of Quaternary Mammals From South America.” Quaternary Science Reviews 55: 103–113. 10.1016/j.quascirev.2012.08.017.

[ece374099-bib-0019] Domingo, L. , R. L. Tomassini , C. I. Montalvo , D. Sanz‐Pérez , and M. T. Alberdi . 2020. “The Great American Biotic Interchange Revisited: A New Perspective From the Stable Isotope Record of Argentine Pampas Fossil Mammals.” Scientific Reports 10: 1680. 10.1038/s41598-020-58575-6. 32005879 PMC6994648

[ece374099-bib-0020] Escobar, S. , A. J. Helmstetter , S. Jarvie , R. Montúfar , H. Balslev , and T. L. Couvreur . 2021. “Pleistocene Climatic Fluctuations Promoted Alternative Evolutionary Histories in *Phytelephas aequatorialis*, an Endemic Palm From Western Ecuador.” Journal of Biogeography 48: 1023–1037. 10.1111/jbi.14055.

[ece374099-bib-0021] Fontes, D. , R. C. Cordeiro , G. S. Martins , et al. 2017. “Paleoenvironmental Dynamics in South Amazonia, Brazil, During the Last 35,000 Years Inferred From Pollen and Geochemical Records of Lago Do Saci.” Quaternary Science Reviews 173: 161–180.

[ece374099-bib-0022] Frederick, L. , A. Brunelle , M. Morrison , P. Crespo , and W. Johnson . 2018. “Reconstruction of the Mid‐Holocene Paleoclimate of the Ecuadorian Andean Páramo at Tres Lagunas, Ecuador.” Holocene 28: 1131–1140. 10.1177/0959683618761547.

[ece374099-bib-0023] Garcia, M. , F. Villalba , L. Araguas‐Araguas , and K. Rozanski . 1998. “The Role of Atmospheric Circulation Patterns in Controlling the Regional Distribution of Stable Isotope Contents in Precipitation: Preliminary Results From Two Transects in the Ecuadorian Andes.” In: Isotope Techniques in the Study of Environmental Change. International Atomic Energy Agency, Vienna, IAEA‐SM‐349/7, 127–140.

[ece374099-bib-0024] Gébelin, A. , C. Witt , M. Radkiewicz , and A. Mulch . 2021. “Impact of the Southern Ecuadorian Andes on Oxygen and Hydrogen Isotopes in Precipitation.” Frontiers in Earth Science 9: 664590. 10.3389/feart.2021.664590.

[ece374099-bib-0025] González‐Guarda, E. , A. P. Loayza , R. A. Segovia , et al. 2025. “Fossil Evidence of Proboscidean Frugivory and Its Lasting Impact on South American Ecosystems.” Nature Ecology & Evolution 9: 1168–1178. 10.1038/s41559-025-02713-8. 40514571

[ece374099-bib-0026] González‐Guarda, E. , A. Petermann‐Pichincura , C. Tornero , et al. 2018. “Multiproxy Evidence for Leaf‐Browsing and Closed Habitats in Extinct Proboscideans (Mammalia, Proboscidea) From Central Chile.” Proceedings of the National Academy of Sciences of the United States of America 115: 9258–9263. 10.1073/pnas.1804642115. 30150377 PMC6140480

[ece374099-bib-0027] González‐Guarda, E. , R. A. Segovia , M. Valenzuela , et al. 2024. “The Extinct *Notiomastodon platensis* (Proboscidea, Gomphoteriidae) Inhabited Mediterranean Ecosystems During the Late Pleistocene in North‐Central Chile (31° S–36° S).” Quaternary Science Reviews 344: 108957. 10.1016/j.quascirev.2024.108957.

[ece374099-bib-0028] Haffer, J. 1969. “Speciation in Amazonian Forest Birds.” Science 165, no. 3889: 131–137. 17834730 10.1126/science.165.3889.131

[ece374099-bib-0029] Hansen, B. C. S. , D. T. Rodbell , G. O. Seltzer , B. León , K. R. Young , and M. Abbott . 2003. “Late‐Glacial and Holocene Vegetational History From Two Sites in the Western Cordillera of Southwestern Ecuador.” Palaeogeography Palaeoclimatology Palaeoecology 194: 79–108. 10.1016/S0031-0182(03)00272-4.

[ece374099-bib-0030] Heusser, L. E. , and N. J. Shackleton . 1994. “Tropical Climatic Variation on the Pacific Slopes of the Ecuadorian Andes Based on a 25,000‐Year Pollen Record From Deep‐Sea Sediment Core Tri 163‐31B.” Quaternary Research 42: 222–225. 10.1006/qres.1994.1072.

[ece374099-bib-0031] Hewitt, G. M. 2004. “Genetic Consequences of Climatic Oscillations in the Quaternary.” Philosophical Transactions of the Royal Society of London. Series B, Biological Sciences 359: 183–195. 10.1098/rstb.2003.1388. 15101575 PMC1693318

[ece374099-bib-0032] Hogg, A. G. , T. J. Heaton , Q. Hua , et al. 2020. “SHCal20 Southern Hemisphere Calibration, 0–55,000 Years Cal BP.” Radiocarbon 62, no. 4: 759–778. 10.1017/RDC.2020.59.

[ece374099-bib-0033] Hooghiemstra, H. , and T. van der Hammen . 1998. “Neogene and Quaternary Development of the Neotropical Rain Forest: The Forest Refugia Hypothesis, and a Literature Overview.” Earth‐Science Reviews 44: 147–183. 10.1016/S0012-8252(98)00027-0.

[ece374099-bib-0034] Hooghiemstra, H. , and T. Van der Hammen . 2004. “Quaternary Ice‐Age Dynamics in the Colombian Andes: Developing an Understanding of Our Legacy.” Philosophical Transactions of the Royal Society of London. Series B: Biological Sciences 359, no. 1442: 173–181. 15101574 10.1098/rstb.2003.1420 PMC1693313

[ece374099-bib-0035] Hostetler, S. W. , and A. C. Mix . 1999. “Reassessment of Ice‐Age Cooling of the Tropical Ocean and Atmosphere.” Nature 399: 673–676. 10.1038/21401.

[ece374099-bib-0036] Iacumin, P. , H. Bocherens , A. Mariotti , and A. Longinelli . 1996. “Oxygen Isotope Analyses of Co‐Existing Carbonate and Phosphate in Biogenic Apatite: A Way to Monitor Diagenetic Alteration of Bone Phosphate?” Earth and Planetary Science Letters 142: 1–6. 10.1016/0012-821X(96)00093-3.

[ece374099-bib-0037] Koch, P. L. , N. C. Tuross , and M. L. Fogel . 1997. “The Effects of Sample Treatment and Diagenesis on the Isotopic Integrity of Carbonate in Biogenic Hydroxylapatite.” Journal of Archaeological Science 24: 417–429. 10.1006/jasc.1996.0126.

[ece374099-bib-0038] Kohn, M. J. 2010. “Carbon Isotope Compositions of Terrestrial C_3_ Plants as Indicators of (Paleo)ecology and (Paleo)climate.” Proceedings of the National Academy of Sciences of the United States of America 107: 19691–19695. 10.1073/pnas.100493310. 21041671 PMC2993332

[ece374099-bib-0039] Levins, R. 1968. Evolution in Changing Environments: Some Theoretical Explorations. Princeton University Press.

[ece374099-bib-0040] Lindsey, E. L. , and E. X. López . 2015. “Tanque Loma, a New Late‐Pleistocene Megafaunal Tar Seep Locality From Southwest Ecuador.” Journal of South American Earth Sciences 57: 61–82. 10.1016/j.jsames.2014.11.003.

[ece374099-bib-0041] Ministerio de Ambiente del Ecuador . 2013. “Sistema de Clasificación de los Ecosistemas del Ecuador Continental.” Subsecretaría de Patrimonio Natural, Quito, Ecuador.

[ece374099-bib-0042] Montilla‐Pacheco, A. , C. I. M. Mora‐Pisco , M. E. D. Durán‐Vasco , and F. R. P. Pastrán‐Calles . 2024. “Contribución al Estudio de la Geografía Climática del Ecuador Continental.” Revista Ciencia UNEMI 17, no. 44: 237–248.

[ece374099-bib-0043] Mothé, D. , and L. Avilla . 2015. “Mythbusting Evolutionary Issues on South American Gomphotheriidae (Mammalia: Proboscidea).” Quaternary Science Reviews 110: 23–35. 10.1016/j.quascirev.2014.12.013.

[ece374099-bib-0044] Mothé, D. , L. S. Avilla , L. Asevedo , et al. 2017. “Sixty Years After ‘The Mastodonts of Brazil’: The State of the Art of South American Proboscideans (Proboscidea, Gomphotheriidae).” Quaternary International 443: 52–64. 10.1016/j.quaint.2016.08.028.

[ece374099-bib-0045] Phillips, D. L. 2012. “Converting Isotope Values to Diet Composition: The Use of Mixingmodels.” Journal of Mammalogy 93: 342–352. 10.1644/11-MAMM-S-158.1.

[ece374099-bib-0046] Prance, G. T. 1982. “A Review of the Phytogeographic Evidences for Pleistocene Climate Changes in the Neotropics.” Annals of the Missouri Botanical Garden 69: 594–624. 10.2307/2399085.

[ece374099-bib-0047] R Core Team . 2023. “R: A Language and Environment for Statistical Computing.” R Foundation for Statistical Computing, Vienna, Austria. https://www.R‐project.org/.

[ece374099-bib-0048] Ramírez‐Barahona, S. , and L. E. Eguiarte . 2013. “The Role of Glacial Cycles in Promoting Genetic Diversity in the Neotropics: The Case of Cloud Forests During the Last Glacial Maximum.” Ecology and Evolution 3: 725–738. 10.1002/ece3.483. 23531632 PMC3605859

[ece374099-bib-0049] Rivals, F. 2019. “MicrowearBivaR: A Code to Create Tooth Microwear Bivariate Plots in R (Version 1).” *Zenodo*. 10.5281/zenodo.2587575.

[ece374099-bib-0050] Rozanski, K. , and L. Araguás Araguás . 1995. “Spatial and Temporal Variability of Stable Isotope Composition of Precipitation Over the South American Continent.” Bulletin de l'Institut Français d'Études Andines 24: 379–390. 10.3406/bifea.1995.1189.

[ece374099-bib-0051] Salati, E. , A. Dall'Olio , E. Matsui , and J. R. Gat . 1979. “Recycling of Water in the Amazon Basin: An Isotopic Study.” Water Resources Research 15: 1250–1258. 10.1029/WR015i005p01250.

[ece374099-bib-0052] Sánchez, B. , J. L. Prado , and M. T. Alberdi . 2004. “Feeding Ecology, Dispersal, and Extinction of South American Pleistocene Gomphotheres (Gomphotheriidae, Proboscidea).” Paleobiology 30: 146–161. 10.1666/0094-8373(2004)030<0146:fedaeo>2.0.co;2.

[ece374099-bib-0053] Semprebon, G. M. , F. Rivals , J. M. Fahlke , W. J. Sanders , A. M. Lister , and U. B. Göhlich . 2016. “Dietary Reconstruction of Pygmy Mammoths From Santa Rosa Island of California.” Quaternary International 406: 123–136. 10.1016/j.quaint.2015.10.120.

[ece374099-bib-0054] Solounias, N. , and G. Semprebon . 2002. “Advances in the Reconstruction of Ungulate Ecomorphology With Application to Early Fossil Equids.” American Museum Novitates 2002, no. 3366: 1–49. 10.1206/0003-0082(2002)366<0001:AITROU>2.0.CO;2.

[ece374099-bib-0055] Stuiver, M. , and P. J. Reimer . 2020. “CALIB 8.0 [Computer Software].” http://calib.org.

[ece374099-bib-0056] Taucare, M. , L. Daniele , B. Viguier , A. Vallejos , and G. Arancibia . 2020. “Groundwater Resources and Recharge Processes in the Western Andean Front of Central Chile.” Science of the Total Environment 722: 137824. 10.1016/j.scitotenv.2020.137824. 32199370

[ece374099-bib-0057] Tejada‐Lara, J. V. , B. J. MacFadden , L. Bermudez , G. Rojas , R. Salas‐Gismondi , and J. J. Flynn . 2018. “Body Mass Predicts Isotope Enrichment in Herbivorous Mammals.” Proceedings of the Royal Society B 285: 20181020. 10.1098/rspb.2018.1020. 30051854 PMC6030519

[ece374099-bib-0058] Tipple, B. J. , S. R. Meyers , and M. Pagani . 2010. “Carbon Isotope Ratio of Cenozoic CO_2_: A Comparative Evaluation of Available Geochemical Proxies.” Paleoceanography 25. 10.1029/2009PA001851.

[ece374099-bib-0059] Tornero, C. , A. Bălăşescu , J. Ughetto‐Monfrin , V. Voinea , and M. Balasse . 2013. “Seasonality and Season of Birth in Early Eneolithic Sheep From Cheia (Romania): Methodological Advances and Implications for Animal Economy.” Journal of Archaeological Science 40: 4039–4055. 10.1016/j.jas.2013.05.013.

[ece374099-bib-0060] Van der Hammen, T. , and H. Hooghiemstra . 2000. “Neogene and Quaternary History of Vegetation, Climate, and Plant Diversity in Amazonia.” Quaternary Science Reviews 19: 725–742. 10.1016/S0277-3791(99)00024-4.

[ece374099-bib-0061] Vogel, J. S. , J. R. Southon , D. E. Nelson , and T. A. Brown . 1984. “Performance of Catalytically Condensed Carbon for Use in Accelerator Mass Spectrometry.” Nuclear Instruments and Methods in Physics Research Section B: Beam Interactions With Materials and Atoms 5: 289–293. 10.1016/0168-583X(84)90529-9.

[ece374099-bib-0062] Vuille, M. , R. S. Bradley , and F. Keimig . 2000. “Climate Variability in the Andes of Ecuador and Its Relation to Tropical Pacific and Atlantic Sea Surface Temperature Anomalies.” Journal of Climate 13: 2520–2535. 10.1175/1520-0442(2000)013<2520:CVITAO>2.0.CO;2.

[ece374099-bib-0063] Xafis, A. , D. Nagel , and K. Bastl . 2017. “Which Tooth to Sample? A Methodological Study of the Utility of Premolar/Non‐Carnassial Teeth in the Microwear Analysis of Mammals.” Palaeogeography, Palaeoclimatology, Palaeoecology 487: 229–240. 10.1016/j.palaeo.2017.09.003.

[ece374099-bib-0064] Ziegler, M. , F. Cruz Jr. , C. Zorro‐Luján , J. Iriarte , and P. Roberts . 2025. “Stable Isotopes Reveal the Northern Tropical Highlands as Refugia for Extinct South American Megafauna.” Research Square Preprint, Version 1. 10.21203/rs.3.rs-6880948/v1.

