# Supplementary material for: Testing the Dry Refuge Model: Paleoecological Insights From Late Pleistocene Gomphotheres in Ecuador
Source: Ecol Evol. 2026 Aug 2;16(8):e74099. doi: 10.1002/ece3.74099 (PMC13429806; doi:10.1002/ece3.74099)
Supplement: Supplementary file 1 — Appendix S1: Description of the current climate and vegetation of the study area, paleoenvironmental background, principles of stable isotope and dental microwear analyses, and assessment of the preservation of the original isotopic composition. [file ECE3-16-e74099-s004.docx]

1. *Current climate and vegetation of the study area*

Topographic features largely control the flow of atmospheric moisture in the Northern Andes (Windhorst et al. 2013). Ecuador has an eastern and a western mountain range, which extend from north to south, influenced by two main sources of precipitation: 1) Air masses originating from the Pacific (Garcia et al. 1998), and 2) Atlantic air masses transported across the Amazon Basin (Windhorst et al. 2013). Also, precipitation regime and atmospheric circulation patterns in Ecuador are governed by three main factors: 1) Altitude of the Andes acting as a weather divide between Pacific air masses from the west and Atlantic air masses from the east (Garreaud et al. 2009), 2) The presence of the Intertropical Convergence Zone (ITCZ) (e.g., Rozanski and Araguas-Araguas 1995), and 3) El Niño-Southern Oscillation (ENSO) (e.g., Rossel and Cardier 2009). The Western Cordillera and coastal areas are affected by western trade winds advecting moisture from the Pacific Ocean (Emck 2007). The climate of the Inter-Andean valley is influenced by moisture sources in the Western Pacific and continental and tropical Atlantic air masses (Vuille et al. 2000). Strong southeasterly trade winds carry moisture from the Amazon and enter the Inter-Andean valley leading to a mixing of moisture with Pacific trade winds (Campozano et al. 2016).

Regarding the vegetation in the study area (Fig. 1) it is possible to describe seven large areas (Ministerio de Ambiente del Ecuador 2013): 1) Dry Coastal Scrubland: it is characterized by a combination of hot and extremely dry conditions, where cacti and other thorny plants are dominant; 2) Coastal Deciduous Forest: compared to evergreen forests, conditions are drier and tree biomass is less dense. Trees are generally less than 20 m tall, with and understory that can be dense and abundant in herbaceous plants; 3) Chocó Tropical Rainforest: where trees thrive under warm, humid conditions. A closed-canopy forests is present, with trees that can reach 30 m in height, and an understory dominated by ferns and plants of the Araceae family; 4) Western Piedmont Forest: present in the western foothills of the Andes mountain Range. It presents a humid and moderately warm climate, with a dominance of palms and trees from the Mimosaceae, Fabaceae, and Burseraceae families. The forest canopy can exceed 30 m, with abundant epiphytic plants (e.g., mosses); 5) Western Montane Forest: it thrives in a temperate climate. The canopy is less than 25 m high, and there is a high abundance of epiphytic plants (e.g., mosses); 6) the more open Western Montane Forest: is restricted to narrow areas between the Mira River basin (near the border with Colombia) and the Chanchán and Chimbo River basins (2° S); and 7) Paramo: its lower altitudinal limit varies between 3,000 and 3,600 m.a.s.l. The dominant vegetation are grasses that form dense stands. The plants in the Paramo are adapted to low temperatures and low water availability. There may also be patches of forest or shrubs.

2. *Paleoenvironmental background*

Available paleoclimatic information from Ecuador is limited to the Middle (781–126 kya) and Late Pleistocene (126–11.7 kya) and the Holocene (11.7 kya–present). This information mainly comes from the analysis of preserved pollen, wood, and phytoliths obtained from soil cores. In general, climate during the Late Pleistocene was colder and drier than current conditions suggesting an increase of open areas because of downslope expansion of paramos and contraction of forests (Heusser and Shackleton, 1994). However, records in the Ecuadorian Amazon have not shown prolonged droughts during the last 94,000 years (Mosblech et al. 2012). Colder conditions were also inferred for the inter Andean valley of northern Ecuador during the Late Pleistocene (Ficcarelli et al. 1997). Contrastingly, climate was only 1–1.5 °C colder during the late Pleistocene than nowadays in the lowlands of the Central Coastal region, suggesting relatively warm conditions (i.e. interstadials) during glacial stages in the referred area (Quiñónez-Macías et al. 2023). These relative warm conditions during the late Pleistocene is supported by the palaeoecological niche modelling of a palm species which supposedly took refuge in the Central Coastal region during the Pleistocene because of stable climatic conditions (Escobar et al. 2021).

During the Holocene, climate was in general warmer and wetter, potentially reducing the extension of open areas because of upslope contraction of paramos and expansion of forests (Heusser and Shackleton 1994). However, there are records at high elevations that suggest that climatic conditions at the paramos from southwestern and southeastern Ecuador during ~15 kya were colder and moister than current conditions (Hansen et al. 2003; Rodríguez and Behling 2012).

Also during the middle Holocene, climatic conditions ameliorated in southwestern, southeastern, and northern Ecuador as warmer and drier conditions have been inferred there (Bakker et al. 2008; Rodríguez and Behling 2012; Frederick et al. 2018), suggesting an upslope contraction of paramos. During the late Holocene at 4–1 kya, paramos from southeastern and northern Ecuador expanded downslope again because of moister conditions (Bakker et al. 2008; Rodríguez and Behling 2012). Once again, paramos from the Inter Andean Valley of northern Ecuador contracted upslope at 0.5 kya (Bakker et al. 2008).

3. *Principles of stable isotopes in mammalian bioapatite*

The interpretation of carbon isotope values found in tooth/bones of medium-to large-bodied herbivorous mammals are based on fractionated values of the plant photosynthetic pathway consumed (Koch, 2007). Plants have two main photosynthetic pathways, C_3_ and C_4_ (Ehleringer and Cerling 2002). C_3_ plants, which fix carbon using the Calvin Cycle, comprise ~85% of terrestrial plant biomass, encompassing most trees, shrubs, and grasses from high-elevation, high latitude, and cool-growing seasons. The δ^13^C values range from –35‰ to –22‰ with mean value of –27‰ (Koch 2007). Values of δ^13^C below –30‰ reflect C_3_ plants from closed-canopy forest environments, whereas values close to the limit of C_3_ plants may be associated with C_3_ grasslands (e.g., –23‰ to –22‰; Kohn 2010). C_4_ plants, in turn, fix carbon using the Hatch-Slack cycle and comprise about 5-10% of the terrestrial plant biomass, including predominantly grasses, herbs and eudicotyledons adapted to arid and high luminosity index environments (Ehleringer and Cerling 2002). The δ^13^C values range from –17‰ to –9‰ and the mean value is –13‰ (Koch 2007).

Thus, the bioapatite carbon isotope composition differs from its source in the diet. This process takes place in ungulate mammals in such a way that δ^13^C values of tooth enamel bioapatite (δ^13^C_enamel_) track the δ^13^C values of consumed plants (δ^13^C_diet_), offset by ~14‰ due to fractionation associated with carbonate equilibria and metabolic processes (Cerling and Harris 1999). Because carbon isotope values vary with the photosynthetic pathways of plants and C_4_ plants, which have much higher carbon isotope values, tend to be more prevalent in open habitats, ranges of δ^13^C values can be estimated for herbivorous mammals in different habitats. Following the classification by Domingo et al. (2012), the ranges of δ^13^C_bioapatite_ values for herbivorous mammals can be estimated for pure C_3_ feeders in different habitats (closed-canopy, –20.5 to –14.5‰; mesic/woodland, –14.5 to –9.5‰; wooded C_3_ grassland to open, arid C_3_ grassland, –9.5 to -6.5‰) and pure C_4_ feeders (–1.5 to –3.5‰). However, the body mass of each taxon should also be considered, since differences have been observed in the standard value of the diet-bioapatite enrichment (Tejada-Lara et al. 2018). Consequently, more precision has recently been achieved regarding habitat ranges for some extinct South American mammals (e.g*.*, Asevedo et al. 2021; Domingo et al. 2020).

The δ^18^O values in mammals show a fractionation of approximately +18‰ between the body water and the bioapatite phosphate (δ^18^O_PO4_). Tooth enamel δ^18^O values of terrestrial mammals are related to the δ^18^O value in body water, which in turn records the body's assimilated and excreted oxygen (Kohn 1996). For a given taxon, the variation in δ^18^O values in body water can be interpreted as being proportional to changes in the isotopic composition of ingested water, which reflects meteoric water influenced by climatic and geographical variables. For a specific region, δ^18^O values in herbivorous mammals depend on each taxon's water requirements. Dietary differences also affect the δ^18^O value of herbivorous mammals. Significant differences have been noted between obligate drinkers, which obtain most water by drinking (and better track changes in δ^18^O values of meteoric water, and in turn in air temperature), and those taxa that do not need to drink (non-obligate drinkers) and get most of their water from plants (and better track humidity/aridity processes suffered by the vegetation) (Kohn 1996).

4. *Principles of dental microwear*

This technique provides insights into the dietary habits of mammals in the days or weeks before death by analyzing microscopic features on the occlusal surfaces of tooth enamel. Dental microwear reflects vegetation availability, habitat, and short-term dietary behavior (Grine 1986).

It uses optical or electron microscopy which allows the identification and quantification of microwear features (striations and depressions) on enamel surfaces (Solounias and Semprebon 2002). This analysis allows individuals to be classified into different dietary categories, for example, frugivores, browsers, grazers, and animals with a mixed diet. In the case of herbivorous mammals, dietary microwear is produced by the plant phytoliths. Also taphonomic processes may also create other marks on the enamel, these can generally be distinguished from those produced in life (King et al. 1999; Uzunidis et al. 2021; Micó et al. 2024a; Micó et al. 2024b).

**Figure S1.** δ^18^O_PO4_ vs. δ^18^O_CO3_ values (‰, V-SMOW) of the Ecuadorian gomphotheres. The data show a slope very close to 1 (0.99) and a high correlation coefficient (R² = 0.96), suggesting equilibrium precipitation of CO_3_^2^⁻ and PO_4_^3^⁻. According to the equation Δ^18^O_CO3_–^18^O_PO4_ = 8.6–9.1‰ (Iacumin et al. 1996), the obtained values (9.3‰) fall within the expected range, indicating that the fossil has retained its original isotopic composition.


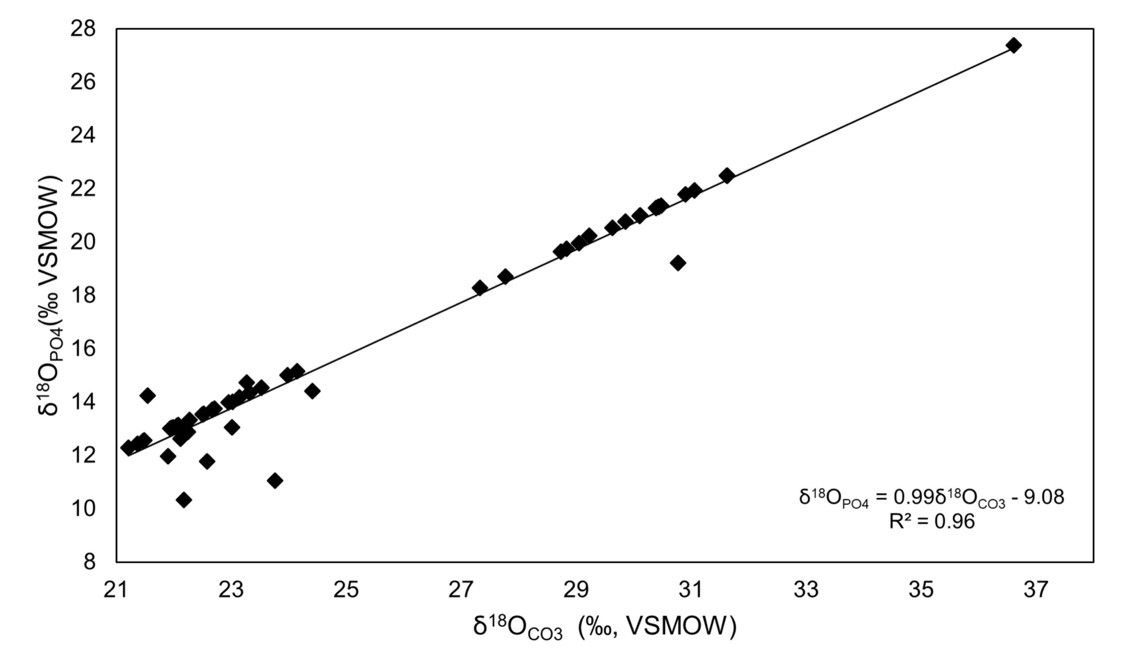


**References**

Asevedo, L., Pansani, T. R., Cordeiro, V. M., Silva-Caminha, S. A. F., Paixão, J. S., Cozzuol, M. A., Dantas, M. A. T., 2021. Diversity of Pleistocene megamammals from southern Amazon, Mato Grosso state, Brazil. J. South. Am. Earth. Sci. 112, 103552. https://doi.org/10.1016/j.jsames.2021.103552.

Bakker, J., Moscol Olivera, M., Hooghiemstra, H., 2008. Holocene environmental change at the upper forest line in northern Ecuador. The Holocene, 18, 877-893. <https://doi.org/10.1177/0959683608093525>.

Campozano, L., Célleri, R., Trachte, K., Bendix, J., and Samaniego, E., 2016. Rainfall and Cloud Dynamics in the Andes: A Southern Ecuador Case Study. Adv. Meteorol. 2016, 3192765. <https://doi.org/10.1155/2016/3192765>.

Cerling, T. E., Harris, J. M., 1999. Carbon isotope fractionation between diet and bioapatite in ungulate mammals and implications for ecological and paleoecological studies. Oecologia. 120, 347-363. <https://doi.org/10.1007/s004420050868>.

Domingo, L., Prado, J.L., Alberdi, M.T., 2012. The effect of paleoecology and paleobiogeography on stable isotopes of Quaternary mammals from South America. Quat. Sci. Rev. 55, 103–113. <https://doi.org/10.1016/j.quascirev.2012.08.017>.

Domingo, L., Tomassini, R.L., Montalvo, C.I., Sanz-Pérez, D., Alberdi, M.T., 2020. The Great American Biotic Interchange revisited: a new perspective from the stable isotope record of Argentine Pampas fossil mammals. Sci. Rep. 10, 1680. https://doi. org/10.1038/s41598-020-58575-6.

Ehleringer, J.R., Cerling, T.E., 2002. C3 and C4 photosynthesis. Encyclopedia of global environmental change. 2, 186–190.

Emck, P., 2007. A Climatology of South Ecuador – with Special Focus on the Major Andean ridge as Atlantic-Pacific Climate divide. Friedrich-Alexander-University of Erlangen-Nuremberg, Germany (Ph.D. Thesis).

Escobar, S., Helmstetter, A. J., Jarvie, S., Montúfar, R., Balslev, H., Couvreur, T. L., 2021. Pleistocene climatic fluctuations promoted alternative evolutionary histories in Phytelephas aequatorialis, an endemic palm from western Ecuador. J. Biogeogr. 48, 1023-1037. https://doi.org/10.1111/jbi.14055.

Ficcarelli, G., Azzaroli, A., Bertini, A., Coltorti, M., Mazza, P., Mezzabotta, C., Moreno-Espinoza, M., Rook, L., Torre, D., 1997. Hypothesis on the cause of extinction of the South American mastodonts. J. South Am. Earth Sci. 10, 29-38. <https://doi.org/10.1016/S0895-9811(97)00003-5>.

García, M., Villalba, F., Araguas-Araguas, L., Rozanski, K., 1998. The Role of Atmospheric Circulation Patterns in Controlling the Regional Distribution of Stable Isotope Contents in Precipitation: Preliminary Results from Two Transects in the Ecuadorian Andes. In Isotope Techniques in the Study of Environmental Change (Vienna: International Atomic Energy Agency), 127–140.

Garreaud, R. D., Vuille, M., Compagnucci, R., Marengo, J., 2009. Present-day South American climate. Palaeogeogr. Palaeoclimatol. Palaeoecol.  281, 180-195. <https://doi.org/10.1016/j.palaeo.2007.10.032>.

Grine, F. E., 1986. Dental evidence for dietary differences in Australopithecus and Paranthropus: a quantitative analysis of permanent molar microwear. J. Hum. Evol. 15, 783-822. https://doi.org/10.1016/S0047-2484(86)80010-0.

Heusser, L. E., Shackleton, N. J., 1994. Tropical climatic variation on the Pacific slopes of the Ecuadorian Andes based on a 25,000-year pollen record from deep-sea sediment core Tri 163-31B. Quat. Res. 42, 222-225. <https://doi.org/10.1006/qres.1994.1072>.

King, T., Andrews, P., Boz, B., 1999. Effect of taphonomic processes on dental microwear. American Journal of Physical Anthropology: The Official Publication of the American Association of Physical Anthropologists, 108, 359-373. https://doi.org/10.1002/(SICI)1096-8644(199903)108:3<359::AID-AJPA10>3.0.CO;2-9

Koch, P.L., 2007. Isotopic study of the biology of modern and fossil vertebrates. In: Michener, R., Lajtha, K. (Eds.), Stable Isotopes in Ecology and Environmental Science, second ed. Blackwell Publishing, Malden, Massachusetts, pp. 99–154. <https://doi.org/10.1002/9780470691854.ch5>.

Kohn, M.J., 1996. Predicting animal δ¹⁸O: Accounting for diet and physiological adaptation. Geochim. Cosmochim. Acta 60, 4811–4829. https://doi.org/10.1016/S0016-7037(96)00240-2.

Kohn, M.J., 2010. Carbon isotope compositions of terrestrial C3 plants as indicators of (paleo)ecology and (paleo)climate. Proc. Natl. Acad. Sci. 107, 19691–19695. <https://doi.org/10.1073/pnas.100493310>.

Micó, C., Blasco, R., Muñoz Del Pozo, A., Jiménez-García, B., Rosell, J., Rivals, F., 2024a. Differentiating taphonomic features from trampling and dietary microwear, an experimental approach. Hist. Biol. 36(4), 760–782. <https://doi.org/10.1080/08912963.2023.2184690>

Micó, C., Blasco, R., Rivals, F., 2024b. Simulating taphonomic processes on teeth: The impact of sediment pressure and thermal alteration on dental microwear. Quat. Sci. Adv. 14, 100195. <https://doi.org/10.1016/j.qsa.2024.100195>

Mosblech, N.A.S., Bush, M.B., Gosling, W.D., Hodell, D., Thomas, L., van Calsteren, P., Correa-Metrio, A., Valencia, B.G., Curtis, J., van Woesik, R., 2012. North Atlantic forcing of Amazonian precipitation during the last ice age. Nat. Geosci. 5(11), 817-820.

Quiñónez-Macías, M., Chunga, K., Toulkeridis, T., Mora-Mendoza, A., Constantine, A., 2023. New Perspectives on the Quaternary Paleogeography of Coastal Ecuador and Its Relationships with Climate Change. Quaternary 6, 41. <https://doi.org/10.3390/quat6030041>.

Rodriguez, F., Behling, H., 2012. Late Quaternary vegetation, climate and fire dynamics, and evidence of early to mid-Holocene Polylepis forests in the Jimbura region of the southernmost Ecuadorian Andes. Palaeogeogr. Palaeoclimatol. Palaeoecol.  350-352, 247-257. <https://doi.org/10.1016/j.palaeo.2012.07.004>.

Rossel, F., and Cadier, E., 2009. El Niño and Prediction of Anomalous Monthly Rainfalls in Ecuador. Hydrol. Process. 23, 3253–3260. <https://doi.org/10.1002/hyp.7401>.

Rozanski, K., Araguás Araguás, L., 1995. Spatial and temporal variability of stable isotope composition of precipitation over the South American continent. Bull. Inst. Fran. Etud. And. 24, 379-390. <https://doi.org/10.3406/bifea.1995.1189>.

Solounias, N., Semprebon, G., 2002. Advances in the reconstruction of ungulate ecomorphology with application to early fossil equids. Am. Mus. Novit. 2002(3366), 1–49. https://doi.org/10.1206/0003-0082(2002)366<0001:AITROU>2.0.CO;2.

Tejada-Lara, J.V., MacFadden, B.J., Bermudez, L., Rojas, G., Salas-Gismondi, R., Flynn, J. J., 2018. Body mass predicts isotope enrichment in herbivorous mammals. Proc. R. Soc. B. 285, 20181020. <https://doi.org/10.1098/rspb.2018.1020>.

Vuille, M., Bradley, R. S., Keimig, F., 2000. Climate Variability in the Andes of Ecuador and its Relation to Tropical Pacific and Atlantic Sea Surface Temperature Anomalies. J. Clim. 13, 2520–2535. [https://doi.org/10.1175/1520-0442(2000)013<2520:CVITAO>2.0.CO;2](https://doi.org/10.1175/1520-0442(2000)013%3c2520:CVITAO%3e2.0.CO;2).

Uzunidis, A., Pineda, A., Jiménez-Manchón, S., Xafis, A., Ollivier, V., Rivals, F. 2021. The impact of sediment abrasion on tooth microwear analysis: an experimental study. Archaeological and Anthropological Sciences, 13(8), 134.
