# Supplementary material for: Testing the Dry Refuge Model: Paleoecological Insights From Late Pleistocene Gomphotheres in Ecuador
Source: Ecol Evol. 2026 Aug 2;16(8):e74099. doi: 10.1002/ece3.74099 (PMC13429806; doi:10.1002/ece3.74099)
Supplement: Supplementary file 3 — Table S1: Summary of stable isotope data (δ13C, ‰V‐PDB) of the molar specimens from the gomphotheres of Ecuador. Number of samples (n), maximum (Max), minimum (Min), mean values and standard deviation (SD). [file ECE3-16-e74099-s003.docx]

**Table S1**. Summary of stable isotope data (δ^13^C, ‰V-PDB) of the molar specimens from the gomphotheres of Ecuador. Number of samples (n), maximum (Max), minimum (Min), mean values and standard deviation (SD).

| **δ^13^C (‰, V-PDB)** | | | | | |
| --- | --- | --- | --- | --- | --- |
| **Locality/Providence** | **n** | **Min** | **Max** | **Mean** | **SD** |
| San Raimundo/Santa Elena (Dry Shrub) (2°24’ S, 80°40’ W) | 4 | –6.76 | –4.45 | –5.74 | 0.96 |
| La Carolina/Santa Elena (Dry Shrub) (2°13' S, 80°55' W) | 13 | –9.36 | –0.78 | –5.52 | 2.47 |
| Pedro Pablo Gómez/Manabí (Western Foothill) (1°37' S, 80°33' W) | 2 | –7.09 | –6.19 | –6.64 | 0.63 |
| Río Chiche/Pichincha (Andean Shrub) (0°12' S, 78°22' W) | 3 | –10.71 | –5.70 | –8.50 | 2.56 |
| Tumbaco/Pichincha (Andean Shrub) (0°15' S, 78°22' W) | 2 | –11.75 | –9.27 | –10.51 | 1.75 |
| La Merced/Pichincha (Andean Shrub) (0°18' S, 78°24' W) | 7 | –10.45 | –4.58 | –7.58 | 2.19 |
| Alangasí-La Merced/Pichincha (Andean Shrub) (0°18' S, 78°24' W) | 1 | –8.01 | –8.01 | –8.01 | - |
| Llano Chico/Pichincha (Andean Shrub) (0° 7' S, 78°25' W) | 3 | –10.94 | –7.05 | –8.41 | 2.20 |
| Calderon/Pichincha (Andean Shrub) (0°15' S, 78°32' W) | 1 | –11.24 | –11.24 | –11.24 | - |
| Alangasí/Pichincha (Andean Shrub) (0°18' S, 78°24' W) | 7 | –14.82 | –7.38 | –10.35 | 2.56 |
| Punín/Chimborazo (Andean Shrub) (1°45' S, 78°39' W) | 7 | –9.71 | –5.10 | –7.48 | 1.83 |
| Quebrada Colorada/Chimborazo (Andean Shrub) (1°46' S, 78°39' W) | 3 | –7.32 | –6.06 | –6.54 | 0.68 |
| Río California, Cuzubamba/Cotopaxi (Eastern Montane) (1° 5' S, 78°41' W) | 1 | –7.36 | –7.36 | –7.36 | - |
