# Supplementary material for: Testing the Dry Refuge Model: Paleoecological Insights From Late Pleistocene Gomphotheres in Ecuador
Source: Ecol Evol. 2026 Aug 2;16(8):e74099. doi: 10.1002/ece3.74099 (PMC13429806; doi:10.1002/ece3.74099)
Supplement: Supplementary file 4 — Table S2: Dental microwear data of gomphothere specimens from the Ecuador. Abbreviations: n, number of specimens; NS, average number of scratches; NP, average number of pits; SWS, scratch width score (0 = only fine scratches, 1 = mixture of fine and coarse scratches, 2 = predominant coarse scratches, 3 = mixture of coarse and hypercoarse scratches, 4 = presence of hypercoarse scratches); PP, presence or absence (0/1) of puncture pits; HC, presence or absence (0/1) of hyper‐coarse scratches; LP, presence or absence (0/1) of large pits; G, presence or absence (0/1) of gouges, and XS, presence or absence (0/1) of cross scratches. [file ECE3-16-e74099-s002.docx]

**Table S2.** Dental microwear data of gomphothere specimens from the Ecuador. Abbreviations: *n*, number of specimens; NS, average number of scratches; NP, average number of pits; SWS, scratch width score (0= only fine scratches, 1= mixture of fine and coarse scratches, 2= predominant coarse scratches, 3= mixture of coarse and hypercoarse scratches, 4= presence of hypercoarse scratches); PP, presence or absence (0/1) of puncture pits; HC, presence or absence (0/1) of hyper-coarse scratches; LP, presence or absence (0/1) of large pits; G, presence or absence (0/1) of gouges, and XS, presence or absence (0/1) of cross scratches.

| **Locality/Province** |  |  | **Dental Microwear** | | | | | | | | |
| --- | --- | --- | --- | --- | --- | --- | --- | --- | --- | --- | --- |
|  | **Code** | | | **NS** | **NP** | **SWS** | **PP** | **HC** | **LP** | **G** | **XC** |
| Calderón/Pichincha | V-1241 | | | 18 | 15 | 2 | 1 | 1 | 1 | 1 | 1 |
| Llano Chico/Pichincha | V-1264 | | | 15 | 11 | 1 | 1 | 1 | 1 | 1 | 1 |
| La Merced/Pichincha | V-6144 | | | 19 | 14 | 1 | 1 | 1 | 1 | 1 | 1 |
| La Merced/Pichincha | V-6143 | | | 16 | 9 | 1 | 1 | 1 | 1 | 1 | 1 |
| La Merced/Pichincha | V-4473 | | | 17 | 10 | 2 | 1 | 1 | 1 | 1 | 1 |
| La Merced/Pichincha | V-6130 | | | 15 | 15 | 2 | 0 | 1 | 1 | 1 | 1 |
| La Merced/Pichincha | V-6132 | | | 19 | 15 | 1 | 1 | 1 | 1 | 1 | 1 |
| Río Chiche/Pichincha | V-1237 | | | 16 | 10 | 1 | 1 | 1 | 1 | 1 | 1 |
| La Carolina/Santa Elena | V-3842 | | | 23 | 12 | 2 | 1 | 1 | 1 | 1 | 1 |
| La Carolina/Santa Elena | V-4824 | | | 16 | 8 | 1 | 1 | 1 | 1 | 1 | 1 |
| La Carolina/Santa Elena | V-3973 | | | 28 | 15 | 1 | 1 | 1 | 1 | 1 | 1 |
| La Carolina/Santa Elena | V-3841 | | | 20 | 12 | 2 | 1 | 1 | 1 | 1 | 1 |
| La Carolina/Santa Elena | V-163 | | | 23 | 10 | 1 | 1 | 1 | 1 | 1 | 1 |
| La Carolina/Santa Elena | V-3843 | | | 17 | 12 | 1 | 1 | 1 | 1 | 1 | 1 |
| San Raimundo/Santa Elena | V-1265 | | | 24 | 11 | 2 | 1 | 1 | 1 | 1 | 1 |
| San Raimundo/Santa Elena | V-1242 | | | 21 | 12 | 2 | 1 | 1 | 1 | 1 | 1 |
| Pedro Pablo Gómez/Manabí | V-4314 | | | 23 | 13 | 1 | 1 | 1 | 1 | 1 | 1 |
| Río California, Cusubamba/Cotopaxi | V-5780 | | | 16 | 13 | 1 | 1 | 1 | 1 | 1 | 1 |
| Quebrada Colorado/Chimborazo | V-4261 | | | 16 | 11 | 1 | 1 | 1 | 1 | 1 | 1 |
| Quebrada Colorado/Chimborazo | V-164 | | | 15 | 9 | 1 | 1 | 1 | 1 | 1 | 1 |
| Quebrada Colorado/Chimborazo | V-1254 | | | 17 | 11 | 1 | 1 | 1 | 1 | 1 | 1 |
