# Supplementary material for: Testing the Dry Refuge Model: Paleoecological Insights From Late Pleistocene Gomphotheres in Ecuador
Source: Ecol Evol. 2026 Aug 2;16(8):e74099. doi: 10.1002/ece3.74099 (PMC13429806; doi:10.1002/ece3.74099)
Supplement: Supplementary file 5 — Table S3: Summary of stable isotope data (δ13C, ‰V‐PDB) and the Estimated Consumed Diet of the gomphotheres specimens from the Ecuador. Number of samples (n), maximum (Max), minimum (Min), mean values and standard deviation (SD). [file ECE3-16-e74099-s006.docx]

**Table S3**. Summary of stable isotope data (δ^13^C, ‰V-PDB) and the Estimated Consumed Diet of the gomphotheres specimens from the Ecuador. Number of samples (n), maximum (Max), minimum (Min), mean values and standard deviation (SD).

| **Estimated Consumed Diet** | | | | | |
| --- | --- | --- | --- | --- | --- |
| **Locality/Providence** | **n** | **Min** | **Max** | **Mean** | **S. Dv.** |
| San Raimundo/Santa Elena (Dry Shrub) (2°24' S, 80°40' W) | 4 | –21.76 | –19.45 | –20.74 | 0.96 |
| La Carolina/Santa Elena (Dry Shrub) (2°13' S, 80°55' W) | 13 | –24.36 | –15.78 | –20.52 | 2.47 |
| Pedro Pablo Gómez/Manabí (Western Foothill) (1°37' S, 80°33' W) | 2 | –22.09 | –21.19 | –21.64 | 0.63 |
| Río Chiche/Pichincha (Andean Shrub) (0°12' S, 78°22' W) | 3 | –25.71 | –20.70 | –23.50 | 2.56 |
| Tumbaco/Pichincha (Andean Shrub) (0°15' S, 78°22' W) | 2 | –26.75 | –24.27 | –25.51 | 1.75 |
| La Merced/Pichincha (Andean Shrub) (0°18' S, 78°24' W) | 7 | –25.45 | –19.58 | –22.58 | 2.19 |
| Alangasí-La Merced/Pichincha (Andean Shrub) (0°18' S, 78°24' W) | 1 | –23.01 | –23.01 | –23.01 | - |
| Llano Chico/Pichincha (Andean Shrub) (0° 7' S, 78°25' W) | 3 | –25.94 | –22.05 | –23.41 | 2.20 |
| Calderon/Pichincha (Andean Shrub) (0°15' S, 78°32' W) | 1 | –26.24 | –26.24 | –26.24 | - |
| Alangasí/Pichincha (Andean Shrub) (0°18' S, 78°24' W) | 7 | –29.82 | –22.38 | –25.35 | 2.56 |
| Punín/Chimborazo (Andean Shrub) (1°45' S, 78°39' W) | 7 | –24.71 | –20.10 | –22.48 | 1.83 |
| Quebrada Colorada/Chimborazo (Andean Shrub) (1°46' S, 78°39' W) | 3 | –22.32 | –21.06 | –21.54 | 0.68 |
| Río California, Cuzubamba/Cotopaxi (Eastern Montane) (1° 5' S, 78°41' W) | 1 | –22.36 | –22.36 | –22.36 | - |
