# Supplementary material for: Testing the Dry Refuge Model: Paleoecological Insights From Late Pleistocene Gomphotheres in Ecuador
Source: Ecol Evol. 2026 Aug 2;16(8):e74099. doi: 10.1002/ece3.74099 (PMC13429806; doi:10.1002/ece3.74099)
Supplement: Supplementary file 6 — Table S4: Summary data of δ13CVPDB, proportional contributions of diet sources (piC3 and piC4 plants) and standardized isotopic niche breadth (B A ) of the Pleistocene gomphotheres from the Ecuador. Number of samples (n), maximum (Max), minimum (Min) and Mean values. [file ECE3-16-e74099-s005.docx]

**Table S4**. Summary data of δ^13^C_VPDB_, proportional contributions of diet sources (*pi*C_3_ and *pi*C_4_ plants) and standardized isotopic niche breadth (*B_A_*) of the Pleistocene gomphotheres from the Ecuador. Number of samples (n), maximum (Max), minimum (Min) and Mean values.

| **Province** | **n** | ***ẟ*^13^C‰ VPDB** | | | ***pi*C_3_** | | | ***pi*C_4_** | | | | **Niche breadth (*BA*)** | | |
| --- | --- | --- | --- | --- | --- | --- | --- | --- | --- | --- | --- | --- | --- | --- |
|  |  | Min | Max | Mean | Min | Max | Mean | | Min | Max | Mean | Min | Max | Mean |
| Santa Elena | 17 | −9.36 | −0.78 | −5.57±2.18 | 0.31 | 0.92 | 0.65±0.16 | | 0.08 | 0.69 | 0.35±0.16 | 0.18 | 1.00 | 0.73±0.22 |
| Manabí | 2 | −7.09 | −6.19 | −6.64±0.63 | 0.69 | 0.76 | 0.72±0.05 | | 0.24 | 0.31 | 0.28±0.05 | 0.58 | 0.74 | 0.66±0.11 |
| Pichincha | 24 | −14.82 | −4.58 | −9.02±2.41 | 0.58 | 1.00 | 0.89±0.17 | | 0 | 0.42 | 0.11±0.17 | 0 | 0.95 | 0.27±0.37 |
| Cotopaxi | 1 | - | - | −7.36 | - | - | 0.78 | | - | - | 0.22 | - | - | 0.53 |
| Chimborazo | 10 | −9.71 | −5.10 | −7.20±1.59 | 0.61 | 0.94 | 0.76±0.11 | | 0.06 | 0.39 | 0.24±0.11 | 0.12 | 0.90 | 0.56±0.28 |
