# Supplementary material for: Testing the Dry Refuge Model: Paleoecological Insights From Late Pleistocene Gomphotheres in Ecuador
Source: Ecol Evol. 2026 Aug 2;16(8):e74099. doi: 10.1002/ece3.74099 (PMC13429806; doi:10.1002/ece3.74099)
Supplement: Supplementary file 7 — Table S5: Statistical summary of stable isotope data (δ18Ometeoric water, ‰V‐SMOW) of the gomphothere specimens from the Ecuador. Number of samples (n), maximum (Max), minimum (Min), mean values and standard deviation (SD). [file ECE3-16-e74099-s007.docx]

**Table S5**. Statistical summary of stable isotope data (δ^18^O_meteoric water_, ‰V-SMOW) of the gomphothere specimens from the Ecuador. Number of samples (n), maximum (Max), minimum (Min), mean values and standard deviation (SD).

| δ**^18^O_meteoric water_ (‰, V-SMOW)** | | | | | |
| --- | --- | --- | --- | --- | --- |
| **Locality/Province** | **n** | **Min** | **Max** | **Mean** | **S. Dv.** |
| San Raimundo/Santa Elena (Dry Shrub) (2°24' S, 80°40' W) | 4 | –10.94 | –2.11 | –5.09 | 4.09 |
| La Carolina/Santa Elena (Dry Shrub) (2°13' S, 80°55' W) | 13 | –4.34 | 4.33 | –1.97 | 2.17 |
| Pedro Pablo Gómez/Manabí (Western Foothill) (1°37' S, 80°33' W) | 2 | –2.70 | –1.46 | –2.08 | 0.88 |
| Río Chiche/Pichincha (Andean Shrub) (0°12' S, 78°22' W) | 3 | –10.20 | –3.88 | –7.64 | 3.32 |
| Tumbaco/Pichincha (Andean Shrub) (0°15' S, 78°22' W) | 2 | –12.06 | –10.37 | –11.22 | 1.20 |
| La Merced/Pichincha (Andean Shrub) (0°18' S, 78°24' W) | 7 | –11.55 | –8.67 | –10.04 | 1.01 |
| Alangasí-La Merced/Pichincha (Andean Shrub) (0°18' S, 78°24' W) | 1 | –9.52 | –9.52 | –9.52 | - |
| Llano Chico/Pichincha (Andean Shrub) (0° 7' S, 78°25' W) | 3 | –11.43 | –10.62 | –11.00 | 0.41 |
| Calderon/Pichincha (Andean Shrub) (0°15' S, 78°32' W) | 1 | –11.45 | –11.45 | –11.45 | - |
| Alangasí/Pichincha (Andean Shrub) (0°18' S, 78°24' W) | 7 | –13.02 | –5.34 | –9.95 | 2.48 |
| Punín/Chimborazo (Andean Shrub) (1°45' S, 78°39' W) | 7 | –13.79 | –9.64 | –11.23 | 1.25 |
| Quebrada Colorada/Chimborazo (Andean Shrub) (1°46' S, 78°39' W) | 3 | –11.72 | –10.16 | –10.90 | 0.79 |
| Río California, Cuzubamba/Cotopaxi (Eastern Montane) (1° 5' S, 78°41' W) | 1 | –10.84 | –10.84 | –10.84 | - |
